# B cell-intrinsic epigenetic modulation of antibody responses by dietary fiber-derived short-chain fatty acids

**DOI:** 10.1038/s41467-019-13603-6

**Published:** 2020-01-02

**Authors:** Helia N. Sanchez, Justin B. Moroney, Huoqun Gan, Tian Shen, John L. Im, Tianbao Li, Julia R. Taylor, Hong Zan, Paolo Casali

**Affiliations:** 1 Department of Microbiology, Immunology & Molecular Genetics, University of Texas Long School of Medicine, UT Health Science Center, San Antonio, TX 78229 USA; 2 Xiangya School of Medicine, Central South University, Changsha, Hunan 410011 China; 3 Department of Molecular Medicine, University of Texas Long School of Medicine, UT Health Science Center, San Antonio, TX 78229 USA

**Keywords:** Immunology, Class switch recombination, Humoral immunity, Somatic hypermutation

## Abstract

Short-chain fatty acids (SCFAs) butyrate and propionate are metabolites from dietary fiber's fermentation by gut microbiota that can affect differentiation or functions of T cells, macrophages and dendritic cells. We show here that at low doses these SCFAs directly impact B cell intrinsic functions to moderately enhance class-switch DNA recombination (CSR), while decreasing at higher doses over a broad physiological range, AID and Blimp1 expression, CSR, somatic hypermutation and plasma cell differentiation. In human and mouse B cells, butyrate and propionate decrease B cell *Aicda* and *Prdm1* by upregulating select miRNAs that target *Aicda* and *Prdm1* mRNA-3′UTRs through inhibition of histone deacetylation (HDAC) of those miRNA host genes. By acting as HDAC inhibitors, not as energy substrates or through GPR-engagement signaling in these B cell-intrinsic processes, these SCFAs impair intestinal and systemic T-dependent and T-independent antibody responses. Their epigenetic impact on B cells extends to inhibition of autoantibody production and autoimmunity in mouse lupus models.

## Abstract (web summary)

Dietary fiber-derived short-chain fatty acids (SCFA) act as histone deacetylase (HDAC) inhibitors on Tregs and innate immune cells, promoting immune tolerance by altering gene expression. Here the authors show that SCFA HDAC inhibitor activity impacts B cell differentiation, antibody responses and antibody-driven autoimmunity.

## Introduction

Epigenetic mechanisms, as influenced by nutritional and environmental factors, including dietary fibers and gut microbiota, maintain and fine-tune immune responses and homeostasis^1–4^. These are critical to health and disease, as they restrict microbial pathogens while restraining immune overactivity. Indeed, immune cells epigenetic dysregulation can result in aberrant immune responses, as in allergy and autoimmunity^5–8^. Gut microbiota, particularly *Clostridia*, a highly polyphyletic class of *Firmicutes*^4,9^, process dietary fibers that are not degraded by the stomach acidic and tryptic environment (resistant fibers) to yield short-chain fatty acids (SCFAs), mostly butyrate (4 carbons)^10^, propionate (3 carbons), and acetate (2 carbons). These SCFAs can modulate mammalian cell functions by serving as energy substrate or by signaling through G-protein-coupled receptors (GPCRs)^1,10–13^. In addition, they directly inhibit histone deacetylases (HDACs) to modulate gene expression^11,14,15^. HDACs remove histone lysine acetyl groups, leading to chromatin condensation and transcriptional silencing. By blocking HDAC-mediated histone deacetylation, butyrate and propionate effectively hyperacetylate histones, thereby enhancing chromatin accessibility and activating gene expression^1,10,16^. Histone acetylation/deacetylation modulates cell gene expression without modifying genomic DNA sequence (epigenetic modification). Other major epigenetic modifications and factors include histone methylation and glycosylation, DNA methylation/demethylation, and noncoding RNAs, such as microRNAs (miRNAs) and long noncoding RNAs (lncRNAs), all of which influence gene transcriptional, post-transcriptional, and post-translational processes^5–7^.

Gut concentration of SCFAs depends on host’s dietary fiber intake, gut bacterial composition, intestinal transit time, and host–microbiota metabolite flux^1,17,18^. Germ-free animals, which have no gut microbiota, show very low levels of gut and periphery SCFAs^15,19,20^, as do mice in a normal environment, but fed a low-fiber or no-fiber diet^20,21^. Dietary fibers and gut microbiota contribute to overall levels of SCFAs. These, particularly butyrate and propionate, impact immune responses locally, in intestinal mucosa and lumen, and systemically, throughout body districts^1–4,10,18^, thereby maintaining a healthy immune homeostasis and prevent allergy and autoimmunity^22,23^. In addition, SCFAs reduce expression of co-stimulatory molecules on antigen-presenting cells, prompting a tolerogenic phenotype, as driven by HDAC inhibition and GPR109A signaling^24^. They can also affect bone marrow hematopoiesis by enhancing generation of dendritic cell (DC) precursors with impaired ability to promote T helper type 2 (Th2) cell effector function^20^. Finally, butyrate and propionate promote thymic production of FOXP3^+^ Tregs and peripheral conversion of T cells into FOXP3^+^ Treg cells^22,25^, through histone (H3) acetylation (by HDAC inhibitory activity) within key regulatory regions of the *Foxp3* locus^15,25^.

SCFAs would mitigate autoimmunity by regulating T cells, DCs, innate lymphoid cells (ILCs), and macrophages^2,3,15,19,20,24,26–29^, increasing anti-inflammatory cytokines, such as TGF-β and IL-10, and inhibiting production of proinflammatory cytokines, such as IL-6, IL-12, IL-17a, IFN-γ, and TNF-α^1,3,30–32^. They can reduce recruitment of eosinophils and allergic cellular infiltration of airways, thereby dampening inflammation and IgE antibody responses^20^. Butyrate and/or propionate may indirectly affect B cells by modulating functions of Treg cells, particularly in autoimmune conditions. Treatment of lupus-prone MRL/*Fas*^*lpr/lpr*^ mice with HDAC inhibitor (HDI) drugs, such as valproic acid (VPA), panobinostat (Farydak), vorinostat (SAHA, Zolinza), or romidepsin (Istodax), reduce autoreactive plasma cell numbers, nephritis, and dampened autoimmunity^33,34^. Other HDIs, such as suberoylanilide hydroxamic acid (SAHA), Trichostatin-A (TSA), and bufexamac, exhert anti-inflammatory effects^22^. As we have shown, VPA, a strong HDAC inhibitor used for epileptic seizures^35^, acts directly on B cells to downregulate *AICDA/Aicda* and *PRDM1/Prdm1* expression in a dose-dependent fashion^7,8,33^. HDAC inhibitory drugs are effective against B lymphocyte lineage malignancies, by inhibiting cell proliferation, survival, and differentiation in an HDAC-class-dependent manner^36,37^. By boosting B-cell metabolism and plasma cell differentiation^12^, SCFAs would potentially support the antibody response, although this contrasts with a large body of evidence emphasizing a potent immunosuppressive activity of gut fiber-derived SCFAs^1,4,9,10,20,22,25,34,38–40^. Thus, whether and how SCFAs impact B-cell differentiation and/or functions remains to be elucidated.

Here, we show that butyrate and propionate act directly on mouse and human B cells to inhibit AID and Blimp1 expression through a B cell-intrinsic, dose-dependent epigenetic HDAC inhibitory activity (not as energy substrate or through GPCR signaling) that leads to upregulation of select miRNAs targeting *Aicda-* and *Prdm1*-3′UTRs. The impact of these SCFAs on B cells in vivo was analyzed by feeding mice a diet-containing fibers (regular chow) or no fiber, together with or without exogenous butyrate and propionate in water. SCFAs concentrations were monitored in the circulation, tissues and feces together with IgD, IgG, IgA, IgE, and bacteria-bound antibodies. Mice fed a no-fiber diet reduced fecal SCFAs, and increased serum and fecal class-switched antibodies. This was reversed by administration of exogenous butyrate and propionate. Through downregulation of B cell AID and Blimp1, these SCFAs inhibited class-switch DNA recombination (CSR), somatic hypermutation (SHM), and plasma cell differentiation in T-dependent and T-independent antibody responses in C57BL/J6 mice, *Tcrβ*^*–/–*^*Tcrδ*^*–/–*^ mice, and *NOD-scid IL2ry*^*null*^ (NSG) mice grafted with purified B cells. The SCFAs B-cell modulatory potency extended to autoantibody responses in lupus-prone MRL/*Fas*^*lpr/lpr*^ and NZB/W F1 mice. Thus, SCFAs derived from gut microbiota-processed dietary fibers modulate antibody and autoantibody responses by impacting directly B-cell-intrinsic epigenetic mechanisms through their HDAC inhibitory activity.

## Results

### Fiber-derived SCFAs reduce local and systemic antibody responses

**Fig. 1:**
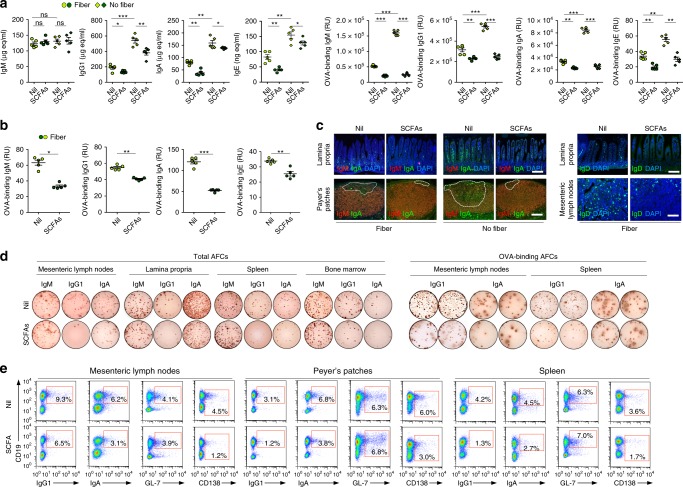
Dietary fibers and SCFAs dampen CSR, plasma cell differentiation and class-switched antibody responses. After weaning, C57BL/6 mice were fed a fiber diet (regular chow) or no-fiber diet. Two weeks later, these mice were started on SCFAs water (SCFAs) or plain water (Nil). All mice were then administered OVA together with CT via intragastric gavage once a week for 4 weeks starting at 8 weeks of age, and killed a week after the last OVA and CT administration. **a** Total and OVA-binding IgM, IgG1, IgA, and IgE concentrations in serum of fiber or no-fiber-fed mice on SCFAs water or plain water, as measured by ELISA. Each symbol represents an individual mouse (*n* = 5 per group, pooled from two experiments). The bars represent the mean ± SD. **b** OVA-binding IgM, IgG1, IgA, and IgE concentrations in the feces of fiber-fed mice on SCFAs water or plain water, as measured by ELISA. Each symbol represents an individual mouse (*n* = 5 per group, pooled from two experiments). **c** IgM, IgA, and IgD-positive cells in the lamina propria, PPs, and MLNs of fiber or no-fiber-fed mice on SCFAs water or plain water as visualized by fluorescence microscopy. **d** ELISPOT analysis of IgM, IgG1, and IgA AFCs in MLNs, PPs, spleen, and bone marrow of fiber-fed mice on SCFAs water or plain water. **e** Proportion of surface CD19^+^IgG1^+^ and CD19^+^IgA^+^ B cells, germinal center B cells (CD19^+^GL-7^+^), and plasmablasts/plasma cells (CD138^+^) in MLNs, PPs, and the spleen of fiber-fed mice given SCFAs water or plain water, as analyzed by flow cytometry. Data in **c**–**e** are representative of three independent experiments. *p *<* 0.05, **p *<* 0.01, ***p *<* 0.001, ns: not significant (unpaired *t* test). Scale bar = 100 μm. The source data are provided in Source Data file.

To address the impact of dietary fiber SCFAs on the antibody response, we fed (after weaning) ten C57BL/6 mice a fiber diet (regular chow, 18% fiber content) and ten mice a no-fiber diet (0% fiber). Two weeks later (at the age of 5 weeks), five mice in each group were started on water-containing SCFAs (20.0 mg/ml tributyrin, 140 mM sodium butyrate, and 150 mM sodium propionate, pH 7.4), and the other five mice on plain water (pH 7.4 and Na^+^ adjusted to match SCFAs water). All mice were then administered ovalbumin (OVA) together with cholera toxin (CT) via intragastric gavage, once a week for 4 weeks. In mice fed fiber diet (regular chow) and plain water, the concentration of butyrate in feces, colon tissue, spleen, and mesenteric lymph nodes (MLNs) were 7.92, 0.46, 0.59, and 0.33 μmol g^–1^, respectively, and those of propionate were 6.28, 0.67, 1.14, and 0.61 μmol g^–1^, respectively (Supplementary Fig. 1a). In circulation, butyrate and propionate were 5–80 μM. SCFAs water to mice on fiber diet increased butyrate and propionate in feces (12.1–23.4 and 13.7–25.9 μmol g^–1^, respectively), colon tissue (1.38; 1.88 μmol g^–1^), spleen (1.43; 2.56 μmol g^–1^), MLNs (1.07; 1.75 μmol g^–1^), and circulation (20–200 μM). These levels were comparable with those in mice or humans fed a fiber or high-fiber diet^20,41^, and led to reduced fecal and circulating levels of total and OVA-specific IgG1, IgA, and IgE (Fig. 1a, b).

**Fig. 2:**
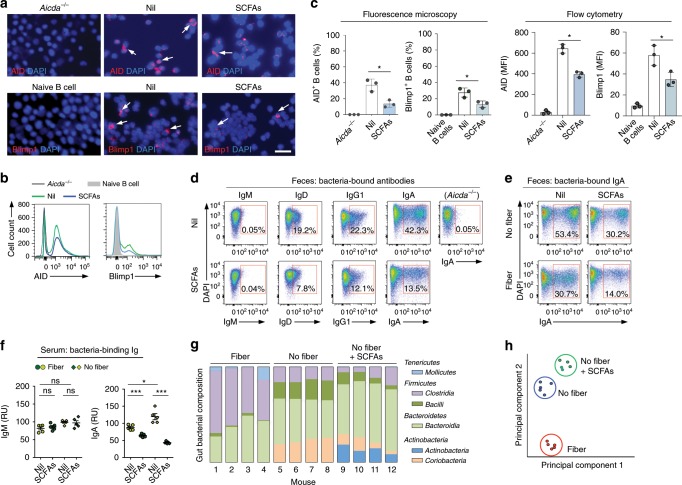
Dietary fibers and SCFAs alter AID and Blimp1 expression, bacteria-bound/binding antibody responses, and gut bacteria composition. C57BL/6 mice fed a fiber or no-fiber diet were on SCFAs water (SCFAs) or plain water (nil) and administered OVA together with CT (same as in Fig. 1). **a**, **b** AID and Blimp1 expression in spleen B cells of these mice, AID expression in spleen B cells of *Aicda*^*-/-*^ mice, and Blimp1 expression in naive B cells of C57BL/6 mice, as analyzed by intracellular staining followed by fluorescence microscopy (**a**) and flow cytometry (**b**). **c** Quantification of AID^+^ and Blimp1^+^ cells in **a** and **b**. *n* = 3 per group. **d** Bacteria-bound IgM, IgD, IgG1, and IgA in the feces of fiber diet-fed C57BL/6 mice on SCFAs water or plain water, collected 1 week after the last OVA and CT administration, as well as bacteria-bound IgA in the feces of unimmunized *Aicda*^*–/–*^ mice fed fiber diet and plain water, as analyzed by flow cytometry. **e** Bacteria-bound IgA in feces of fiber and no-fiber diet-fed mice on SCFAs water or plain water as analyzed by flow cytometry. **f** Concentration of bacteria-binding IgM and IgA in the serum as measured by ELISA; each symbol represents an individual mouse (*n* = 5 per group, pooled from two experiments). The bars represent the mean ± SD. **g** Relative abundance of gut bacteria classes (> 1% in any sample group; fiber, no-fiber, and no-fiber + SCFAs) in the mice (*n* = 4 per group, pooled from two experiments) as determined by high-throughput 16s rRNA gene miSeq sequencing. **h** Principal component analysis of gut bacterial composition in these three groups of mice on different diets. Data in **a**, **b**, **d**, and **e** are repr**e**sentative of three independent experiments. *p *<* 0.05, ***p *<* 0.001 values, ns: not significant (unpaired *t* test). Scale bar = 100 μm. The source data are provided in Source Data file.

The mice fed no-fiber diet and plain water displayed a low level of butyrate and propionate in feces (butyrate, 1.1–1.8 μmol g^–1^; propionate, 1.1–2.1 μmol g^–1^) and high levels of total and OVA-specific IgG1, IgA, and IgE, which upon administration of SCFAs were brought down to the levels observed in fiber diet-fed mice on SCFAs water (Supplementary Fig. 1a; Fig. 1a, b)—acetate was not used in these in vivo experiments, as this SCFA does not display a significant HDAC inhibitory activity^11,14,15^ and it did not alter *Aicda* or *Prdm1* expression and CSR or plasma cell differentiation (Supplementary Fig. 1b–d). When added to the fiber diet, exogenous butyrate and propionate further decreased circulating class-switched antibodies and local (intestinal) IgA- and IgD-expressing cells in Peyer’s patches (PPs), lamina propria (LP), and/or MLNs, as well as the total IgG1 and IgA but not IgM antibody-forming cells (AFCs) in LP, MLNs, spleen, and bone marrow. The local and systemic reduction in AFCs was associated with decreased numbers of IgA^+^ and IgG1^+^ B cells, as well as CD138^+^ plasmablasts/plasma cells, but not germinal center (GL-7^+^) B cells in PPs, MLNs, and spleen of SCFA-treated fiber-fed mice (Fig. 1c–e; Supplementary Fig. 2). By contrast, the no-fiber diet increased intestinal and systemic class-switched B cells, AFCs, and antibody levels, which were reduced by administration of exogenous SCFAs. The reduction of local and systemic class-switched B cells, AFCs, and class-switched antibodies by SCFAs reflected an inhibition of AID and Blimp1 expression (Fig. 2a–c; Supplementary Fig. 2), and was associated with increased fecal butyrate (12.7 vs.1.4 μmol g^–1^) and propionate (14.2 vs. 1.5 μmol g^–1^) in mice fed a no-fiber or fiber diet in conjunction with exogenous SCFAs (Supplementary Fig. 1a). Thus, butyrate and propionate from fermentation of dietary fibers reduced B cell AID and Blimp1, class-switched B cells, AFCs, and class-switched antibodies at local (intestinal) and systemic levels.

### SCFAs dampen systemic and fecal anti-bacteria antibodies

Exogenous SCFAs further reduced B-cell expression of AID and Blimp1 as well as fecal bacteria-bound IgD, IgG1, and IgA but not the (low) level of bacteria-bound IgM (virtually no bacteria-bound IgA were detected in *Aicda*^*-/-*^ mice) in mice on fiber diet. In the absence of exogenous SCFAs, mice on fiber diet (regular chow) reduced fecal bacteria-bound IgA and circulating bacteria-binding IgA in fiber vs. no-fiber-fed mice, without altering circulating levels of bacteria-binding IgM (Fig. 2a–f; Supplementary Fig. 2). To address the influence of dietary fibers and SCFAs on gut bacterial composition, we performed high-throughput sequencing of fecal bacterial 16S rRNA genes. The fiber or no-fiber diet with or without exogenous SCFAs resulted in comparable gut microbial composition in all four mice of each diet group (Fig. 2g). *Firmicutes Clostridia* and *Firmicutes Bacilli* as well as *Bacteriodetes Bacteroidia*, which are major SCFAs producers^9^, were present in all three dietary groups, albeit at different frequencies. These were conditioned mainly by the presence or absence of dietary fibers, as exogenous SCFAs diversified gut bacterial composition only marginally (principal component analysis, Fig. 2h).

### SCFAs dampen hypermutated and class-switched antibody responses

**Fig. 3:**
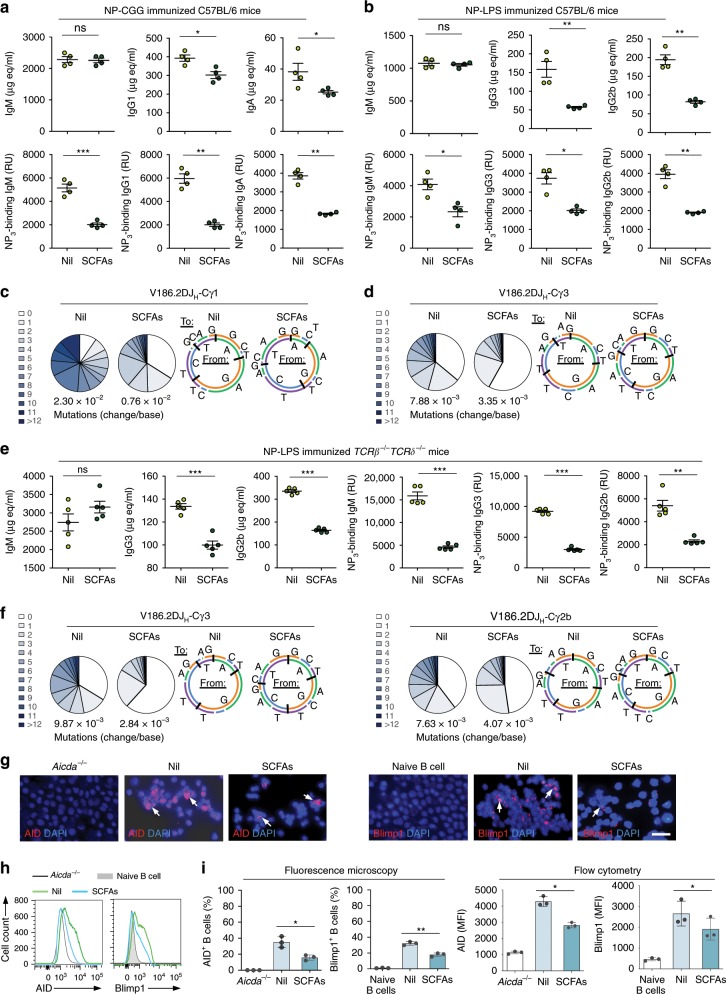
SCFAs reduce class-switched and hypermutated T-dependent and T-independent antibody responses. **a**–**d** C57BL/6 mice fed fiber diet on SCFAs water (SCFAs) or plain water (Nil) starting at the age of 5 weeks, and i.p. injected with NP_16_-CGG (in Alum) or NP-LPS (in PBS) at the age of 8 weeks. The mice were boost-injected 3 weeks later with NP_16_-CGG or NP-LPS, respectively, and killed a week after the boost injection. **a**, **b** Titers of total and NP_3_-binding serum IgM, IgG1, and IgA in NP_16_-CGG injected C57BL/6 mice (**a**), as well as total and NP_3_-binding serum IgM, IgG3, and IgG2b titers in NP-LPS injected C57BL/6 mice (**b**), as measured by ELISA (*n* = 4 per group, pooled from two experiments). Bars represent the mean ± SD. **c**, **d** Somatic point mutations in the V_186.2_ regions from V_186.2_DJ_H_-Cγ1 transcripts of NP_16_-CGG injected C57BL/6 mice (**c**), or from V_186.2_DJ_H_-Cγ3 transcripts of the NP-LPS injected C57BL/6 mice (**d**). Sequence data were pooled from three mice in each group. Pie charts depict the proportions of sequences that carry different numbers of point mutations; listed below the pie charts is the overall mutation frequency (change/base). Donut charts depict the spectrum of point mutations. **e**–**h** *Tcrβ*^*–/–*^*Tcrδ*^*–/–*^ mice on SCFAs water or plain water were injected with NP-LPS. Total and NP_3_-specific serum IgM, IgG3, and IgG2b titers were measured by ELISA (*n* *=* 5 per group) (**e**). Somatic point mutations in the V_186.2_ region of V_186.2_DJ_H_-Cγ3 and V_186.2_DJ_H_-Cγ2b transcripts. Sequence data were pooled from three mice in each group (**f**). AID and Blimp1 expression in spleen B cells of these mice, AID expression in spleen B cells of *Aicda*^*-/-*^ mice, and Blimp1 expression in naive B cells of C57BL/6 mice, as analyzed by intracellular staining followed by fluorescence microscopy (**g**), and flow cytometry (**h**). Data in **g** and **h** are representative of three independent experiments. **i** Quantification of AID^+^ and Blimp1^+^ cells in **h** and **g**. *n* = 3 per group. *p *<* 0.05, **p *<* 0.01, ***p *<* 0.001 (unpaired *t* test). Scale bar = 10 μm. The source data are provided in Source Data file.

In addition to dampening class-switched IgG1, IgA, and IgE to OVA, SCFAs reduced circulating OVA-binding IgM titers, but not total IgM. We reasoned that the reduced OVA-binding IgM but not total IgM resulted from inhibition of SHM by butyrate and propionate and, consequently, impaired antibody affinity maturation. To address the impact of SCFAs on SHM, we injected eight C57BL/6 mice with NP_16_-CGG i.p., and fed four of such mice SCFAs water and the other four plain water. SCFAs did not affect the overall level of total IgM, but reduced total IgG and IgA as well as (high affinity) NP_3_-binding IgM, IgG, and IgA, likely a reflection of the 66% decrease in somatic mutations of the recombined V_186.2_ gene segment, which encodes the NP-binding site (Fig. 3a, c).

As a first step toward addressing the SCFAs-mediated T-independent, and possibly B-cell-intrinsic, modulation of the antibody response, we fed four C57BL/6 mice SCFAs water and another four plain water, and then i.p. injected all of them with NP-LPS. SCFAs curtailed the T-independent class-switched and high-affinity NP_3_ (exclusively IgG3 and IgG2b) antibody response, with a significantly lower mutational load in recombined V_186.2_ gene segments, albeit of different magnitude from that of the T-dependent response. This occurred without changes in proportion of B220^+^ B cells, CD3^+^, CD4^+^, and CD8^+^ T cells, or B-cell proliferation, viability, and germinal center formation (Fig. 3b, d; Supplementary Fig. 3a, b, e).

To further show that SCFAs can impair the maturation of the antibody response independently of T cells, we analyzed the anti-NP_3_ IgG3 and IgG2b response in ten *Tcrβ*^*–/–*^*Tcrδ*^*–/–*^ mice (devoid of both αβ^+^ and γδ^+^ T cells^42^). These mice were divided into two groups of five each, which were fed plain or SCFAs water, and then i.p. injected with NP-LPS. The *Tcrβ*^*–/–*^*Tcrδ*^*–/–*^ mice fed plain water mounted a robust class-switched and hypermutated antibody response to NP. Such a response was reduced by SCFAs in term of total and high-affinity NP_3_-specific IgG3 and IgG2b, likely reflecting the defective CSR and decreased somatic point mutations in V_186.2_ segment of V_186.2_DJ_H_-Cγ3 and V_186.2_DJ_H_-Cγ2b transcripts (by 71% and 47%). The SCFA-mediated inhibition of CSR/SHM was directly associated with reduced B-cell expression of AID and Blimp1, and normal germinal center formation (Fig. 3e–i; Supplementary Figs. 3c, 4, 5).

**Fig. 4:**
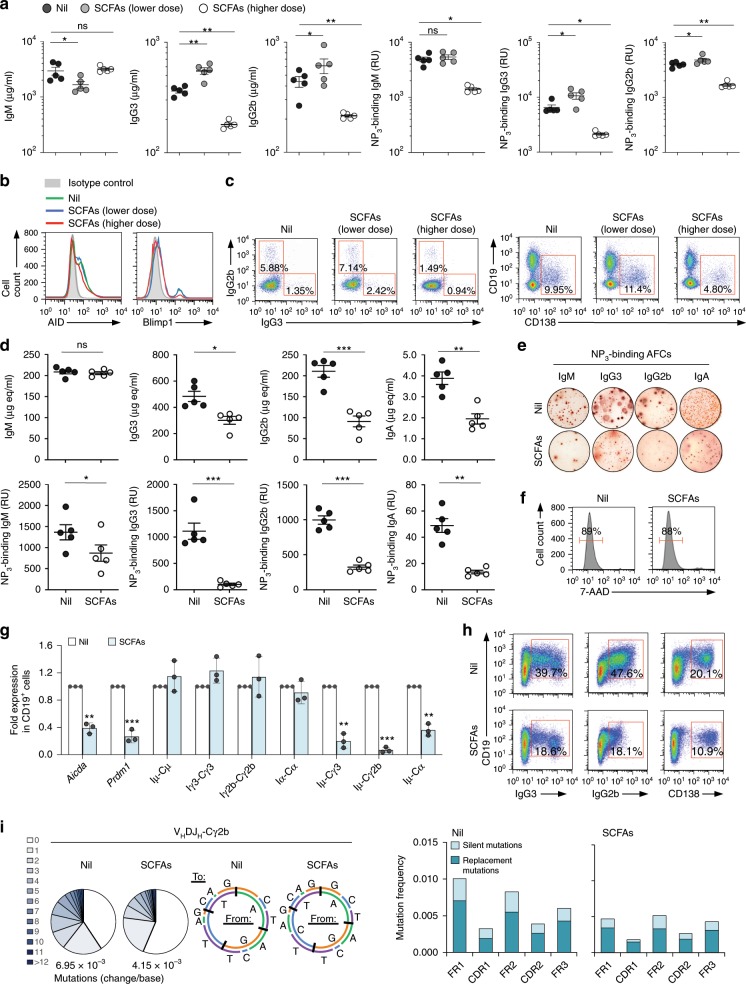
Dose-dependent and B-cell-intrinsic modulation of *Aicda* and *Prdm1* expression, CSR, SHM and plasma cell differentiation by SCFAs in vivo. **a**–**c** C57BL/6 mice on plain water (Nil), SCFAs “lower dose” water (pH = 7.4, 20 mM sodium butyrate, and 30 mM sodium propionate) or SCFAs “higher dose” water (pH = 7.4, 20 mg ml^−1^ tributyrin emulsion, 140 mM sodium butyrate and 150 mM sodium propionate, SCFAs higher dose. The SCFAs water is referred here as SCFAs “higher dose” as opposed to the “lower dose” used in these experiments for the sake of clarify) were i.p. injected with NP-LPS. **a** Total and NP_3_-binding IgM, IgG2b, and IgG3 induced by low dose of SCFAs, as measured by ELISA. Each symbol represents an individual mouse (*n* = 4–5 per group, pooled from two experiments). Bars represent mean ± SD. **b** AID and Blimp1 expression in spleen B cells from the same mice, as analyzed by intracellular staining followed flow cytometry. **c** IgG3^+^, IgG2b^+^ B cells, and CD19^low^CD138^+^ plasmablasts/plasma cells in the spleen, as analyzed by flow cytometry. Data in **b** and **c** are representative of three independent experiments. **d**–**i** NSG/B mice on plain water or SCFAs water (pH = 7.4, 20 mg ml^−1^ tributyrin emulsion, 140 mM sodium butyrate, and 150 mM sodium propionate) were injected with NP-LPS. **d** Titers of total and NP_3_-binding serum IgM, IgG3, IgG2b, and IgA, as analyzed by ELISA. Each symbol represents an individual mouse (*n* = 5 per group from one experiments). **e** NP_3_-binding IgM, IgG3, IgG2b, and IgA AFCs in spleen as analyzed by ELISPOT. **f** Viable (7-AAD^−^) spleen CD19^+^ B cells as analyzed by flow cytometry. **g** *Aicda, Prdm1*, germline I_H_-C_H_, and post-recombination Iμ-C_H_ transcripts, as analyzed by qRT-PCR and normalized to *Gapdh*. Data are ratios to B cells cultured with nil (set as 1; means ± SEM of three independent experiments). **h** Proportion of IgG3^+^ and IgG2b^+^ B cells, and CD138^+^ plasmablasts/plasma cells in the spleen, as analyzed by flow cytometry. **i** Somatic point mutations in the V_H_ region of V_H_DJ_H_-Cγ2b transcripts. Mutations are in pooled sequences from two out of five mice per group. Pie charts depict the proportions of sequences that carry different numbers of point mutations; listed below the pie charts is the overall mutation frequency. Donut charts depict the spectrum of point mutations. Histograms depict frequencies of silent and replacement mutations in FR and CDR regions. *p < 0.05, **p < 0.01, ***p < 0.001, ns: not significant (unpaired *t* test). The source data are provided in Source Data file.

Having shown that fiber-derived SCFAs inhibit antibody responses at intestinal and systemic levels, we addressed the suggestion that at a low concentration, SCFAs increase IgG and IgA responses^12^. To this end, we fed five C57BL/6 mice SCFAs water, and five mice water containing a low dose of SCFAs (20 mM sodium butyrate and 30 mM, comparable with what used by Kim et al.^12^). A third group of five mice were fed plain water. All mice were injected with NP-LPS. While SCFA water significantly reduced total and NP_4_-binding IgG2b and IgG3, B cell AID and Blimp1 expression, IgG3^+^ and IgG2b^+^ B cells, as well as plasma cells, low- dose SCFA water led to a moderate increase in AID and Blimp1 expression, IgG2b^+^ and IgG3^+^ B cells, and CD19^low^CD138^+^ plasma cells, as well as circulating total and NP-binding IgG2b and IgG3 (Fig. 4a–c).

To further address the direct B-cell-intrinsic SCFA-mediated impairment of the antibody response in vivo, we developed “NSG/B” mice by engrafting immuno-deficient NOD-*scid IL2rγ*^*null*^ (NSG) mice^43^, which lack T cells, B cells, NK cells, functional DCs, and macrophages, with highly purified C57BL/6 spleen B cells. We segregated ten NSG/B mice into two groups, each of five mice. One group was fed SCFAs water and the other plain water, all of them were then i.p. injected with NP-LPS. All NSG/B mice fed plane water made IgM, IgG2b, IgG3, and IgA antibodies to NP_3_. However, the NSG/B mice given SCFAs water reduced total and NP_3_-binding IgG3, IgG2b and IgA, as well as NP_3_-binding but not total IgM, likely reflecting the reduced number of spleen NP_3_-specific IgM-, IgG3-, IgG2b-, and IgA AFCs, in the absence of altered B-cell viability or number (Fig. 4d–f). This was underpinned by reduced *Aicda* and *Prdm1* expression and decreased post-recombination Iμ-Cγ3, Iμ-Cγ2b, and Iμ-Cα transcripts, and was concomitant with reduced numbers of IgG3^+^ and IgG2b^+^ B cells, as well as CD138^+^ plasmablasts/plasma cells; exogenous SCFAs also reduced (by 40.3%) the mutational load in V_H_DJ_H_-Cγ2b transcripts (Fig. 4g–i). Thus, SCFAs can impair the maturation of a class-switched high-affinity antibody response by downregulating *Aicda* and *Prdm1* expression, thereby dampening CSR/SHM and plasma cell differentiation, in a T-cell-, DC-, NK cell-, and macrophage-independent, and B-cell-intrinsic fashion.

### Dose-dependent B cell *AICDA/Aicda* and *PRDM1/Prdm1* inhibition by SCFAs

**Fig. 5:**
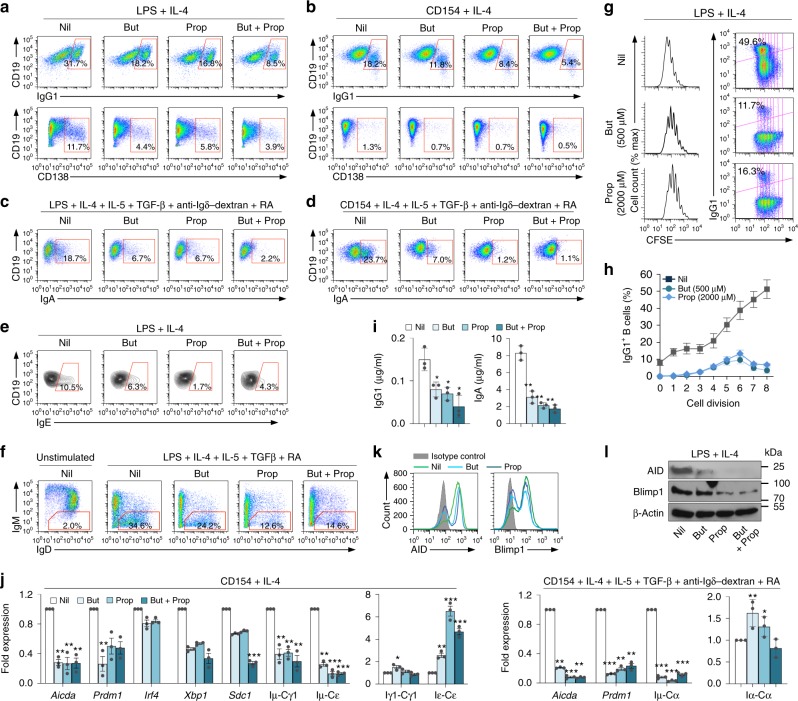
SCFAs inhibit *Aicda* and *Prdm1* expression, and reduce CSR and plasma cell differentiation in mouse B cells. **a**–**f** Mouse B cells were stimulated in the presence of Nil, butyrate (500 μM, But), propionate (2000 μM, Prop), or butyrate (500 μM) plus propionate (2000 μM). Proportions of surface CD19^+^ IgG1^+^ B cells and CD138^+^ plasmablasts/plasma cells (**a**, **b**), surface CD19^+^ IgA^+^ B cells (**c**, **d**), intracellular CD19^+^ IgE^+^ B cells (**e**), and surface CD19^+^ IgM^–^ IgD^+^ B cells (**f**) as analyzed by flow cytometry 96 h after stimulation. **g**, **h** CD19^+^ B cells were labeled with CFSE and stimulated for 96 h with LPS plus IL-4 in the presence of nil, butyrate (500 μM), or propionate (2000 μM). CFSE intensity and surface IgG1 expression as analyzed by flow cytometry. Progressive left shift of fluorescence intensity indicates CD19^+^ B-cell division. Data are representative of three independent experiments yielding comparable results (**g**). Proportion of surface IgG1^+^ B cells at each cell division. Data are mean and SE from three independent experiments (**h**). **i** Titers of IgG1 and IgA in culture fluids as analyzed by ELISA. Data are from three independent experiments (mean and SE). **j** *Aicda, Prdm1*, *Irf4, Xbp1, Sdc1*, post-recombination Iμ-Cγ1, Iμ-Cε, and Iμ-Cα transcripts as well as germline Iγ1-Cγ1, Iε-Cε, and Iα-Cα transcripts in B cells stimulated with LPS plus IL-4, or CD154 plus IL-4, IL-5, TGF-β, anti-δ mAb-dextran in the presence of butyrate, propionate, or butyrate plus propionate for 60 h, as analyzed by qRT-PCR and normalized to *Gapdh* transcripts. Data are ratios to stimulated B cells cultured with nil (set as 1; means ± SEM of three independent experiments). **k**, **l** B cells were stimulated with LPS plus IL-4 in the presence of nil, butyrate, propionate, or butyrate plus propionate for 72 h. The B-cell expression of AID and Blimp1 as determined by intracellular staining and flow cytometry (**k**). AID, Blimp1, and β-Actin proteins, as detected by immunoblotting (**l**). Data are one representative of three independent experiments yielding comparable results. *p *<* 0.05, **p *<* 0.01, ***p *<* 0.001 (unpaired *t* test). The source data are provided in Source Data file.

To define the direct and B-cell-intrinsic impact of SCFAs on *Aicda* and *Prdm* expression, we induced mouse B cells to undergo CSR to IgD, IgG1, IgA, IgE, and plasma cell differentiation in the presence of nil, butyrate (50–500 μM), propionate (50–2000 μM), or butyrate (500 μM) plus propionate (1000 μM). The butyrate and propionate concentrations used in these in vitro experiments were within the range of those detected in the spleen, colon, and MLNs (Supplementary Fig. 1a). Butyrate and propionate decreased CSR to all Ig isotypes and plasma cell differentiation in a dose-dependent fashion without altering B-cell viability or proliferation, as exemplified by the lower proportion of class-switched IgG1^+^ B cells in each round of cell division (Fig. 5a–h and Supplementary Figs. 3d, f, 6, 7). The inhibition of CSR and plasma cell differentiation by butyrate (500 μM) and propionate (2000 μM) was further confirmed by decreased IgG1 and IgA secretion, decreased post-recombination Iμ–Cγ1, Iμ–Cα and Iμ–Cε transcripts, reduced *Aicda* and *Prdm1* transcripts, and AID and Blimp1 proteins. Germline Iμ–Cμ, Iγ1–Cγ1, Iα-Cα, and Iε–Cε transcripts, which are also involved in CSR, were not reduced, rather increased by butyrate and propionate in a dose-dependent fashion, likely due to the HDAC inhibitory activity of such SCFAs (Fig. 5i–l; Supplementary Fig. 8a). To define whether SCFA acetate also affects CSR and plasma cell differentiation, we stimulated B cells with LPS and IL-4, in the presence of increasing doses of acetate or acetate together with butyrate or propionate. As predicted, unlike butyrate and propionate, acetate did not alter *Aicda* and *Prdm1* expression or CSR to IgG1 and plasma cell differentiation (Supplementary Fig. 1b, c), nor did it affect *Aicda* and *Prdm1* inhibition by butyrate and propionate (Supplementary Fig. 9). To define whether, like what we found in vivo, low and higher SCFAs concentrations modulate differentially CSR and plasma cell differentiation, we stimulated B cells with LPS plus IL-4 in the presence of increasing concentrations of SCFAs (butyrate, 50–800 μM; propionate, 50–3200 μM). At concentrations comparable to or higher than those in colon and lymphoid tissues of mice fed a fiber diet and plain water (butyrate, ≥ 400 μM; propionate, ≥ 800 μM), butyrate and propionate inhibited *Aicda* expression in a dose-dependent fashion. By contrast, at low concentrations (butyrate, 50, 100, or 200 μM; propionate, 50, 100, 200, or 400 μM), these SCFAs moderately increased *Aicda* expression (Supplementary Fig. 8a).

**Fig. 6:**
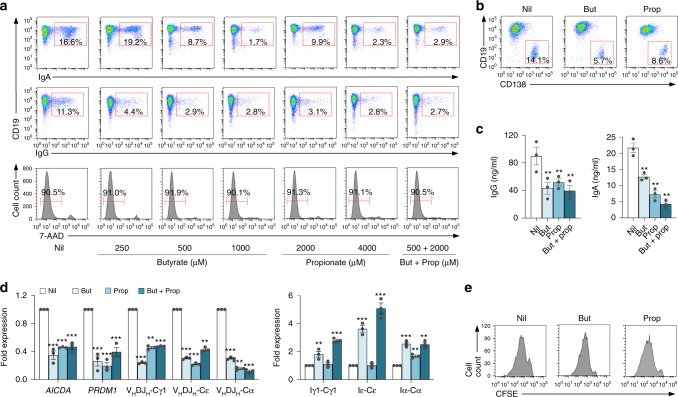
Butyrate and propionate inhibit *AICDA* and *PRDM1* expression, and reduce CSR and plasma cell differentiation in human B cells. **a** Human B cells purified from PBMCs of healthy subjects were stimulated with CD154 plus IL-4 and IL-21, in the presence of nil, butyrate (250, 500, or 1000 μM), propionate (2000 or 4000 μM), or butyrate (500 μM) plus propionate (2000 μM). The proportions of CD19^+^ IgA^+^ and CD19^+^ IgG^+^ as well as viable (7-AAD^–^) CD19^+^ B cells were analyzed 120 h post stimulation by flow cytometry. Data are representative of three independent experiments yielding comparable results. **b**–**d** Human B cells were stimulated with CD154 plus IL-4 and IL-21, in the presence of nil, butyrate (500 μM), propionate (2000 μM), or butyrate (500 μM) plus propionate (2000 μM). CD19^lo^CD138^+^ plasma cells were analyzed 120 h post stimulation by flow cytometry (**b**). IgG and IgA titers in culture fluids of these B cells were analyzed 120 h post stimulation by ELISA. Data are from three independent experiments (mean and SE) (**c**). Expression of *AICDA* and *PRDM1*, germline Iγ1-Cγ1, Iα-Cα, and Iε-Cε, as well as mature V_H_DJ_H_-Cγ1, V_H_DJ_H_-Cε, and V_H_DJ_H_-Cα transcripts were analyzed 72 h post stimulation by qRT-PCR and normalized to the expression of *HRPT*. Data are ratios to stimulated B cells cultured with nil (set as 1; means ± SEM of three independent experiments) (**d**). **e** Human B cells were labeled with CFSE and stimulated with CD154 plus IL-4 and IL-21 in the presence of nil, butyrate (500 μM), or propionate (2000 μM) for 96 h. CFSE intensity were analyzed by flow cytometry. Progressive left shift of fluorescence intensity indicates CD19^+^ B-cell division. Data are representative of three independent experiments yielding comparable results. **p *<* 0.01, ***p *<* 0.001 (unpaired *t* test). The source da*t*a are provided in Source Data file.

As they did in mouse B cells, butyrate and propionate inhibited CSR to IgG, IgA, and IgE, as well as plasma cell differentiation in human B cells in a dose-dependent fashion. They reduced the number of surface IgG^+^ and IgA^+^ B cells, decreased secreted IgG and IgA as well as mature post-recombination V_H_DJ_H_-Cγ1, V_H_DJ_H_-Cα, and V_H_DJ_H_-Cε transcripts, without altering B-cell viability or proliferation. This was concomitant with downregulation of *AICDA* and *PRDM1* transcripts, and increased germline Iγ1-Cγ1, Iα-Cα, and Iε-Cε transcripts, likely due to the HDAC inhibitory activity of butyrate and propionate (Fig. 6a–e; Supplementary Fig. 7d). Thus, SCFAs directly impact mouse and human B cells to decrease *Aicda*/*AICDA* and *Prdm1/PRDM1* expression, thereby reducing in a dose-dependent fashion CSR to IgG, IgA, and IgE as well as plasma cell differentiation.

### SCFAs dampen *Aicda*/*Prdm1* through HDAC inhibitory activity

**Fig. 7:**
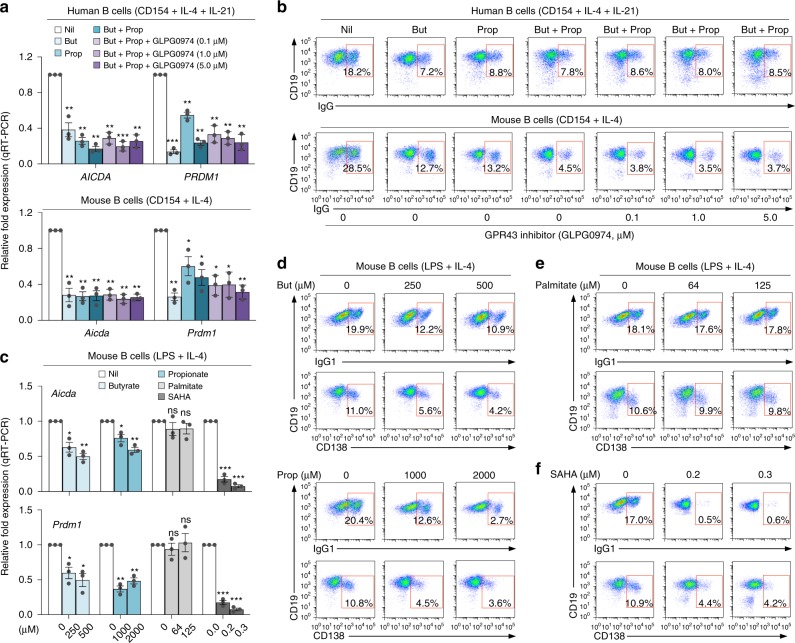
SCFAs inhibit *AICDA/Aicda* and *PRDM1/Prdm1* expression, CSR, and plasma cell differentiation through their HDAC inhibitory activity. **a**, **b** Human and mouse B cells were stimulated with CD154 plus IL-4 and IL-21, or CD154 plus IL-4, respectively, in the presence of nil, butyrate (500 μM), propionate (2000 μM), butyrate (500 μM) plus propionate (2000 μM) alone, or butyrate (2000 μM) plus propionate (2000 μM) together with increasing doses of GPR43 inhibitor GLPG0974. Human B cell *AICDA* and *PRDM1* transcripts as well as mouse B cell *Aicda* and *Prdm1* transcripts were analyzed 72 h post stimulation by qRT-PCR and normalized to *HRPT* and *Gapdh* expression, respectively. Data are ratios to stimulated B cells cultured with nil (set as 1; means ± SEM of three independent experiments) (**a**). Proportion of CD19^+^IgG^+^ B cells were analyzed 120 h post stimulation by flow cytometry. Data are representative of three independent experiments yielding comparable results (**b**). **c**–**f** Mouse B cells were stimulated with LPS plus IL-4, in the presence of increasing doses of butyrate, propionate, palmitate, and SAHA. Expression of *Aicda* and *Prdm1* transcripts were analyzed 72 h post stimulation by qRT-PCR and normalized to *Gapdh* expression. Data are ratios to stimulated B cells cultured with nil (set as 1; means ± SEM of three independent experiments) (**c**). The proportion of CD19^+^ IgG1^+^ B cells and CD138^+^ plasmablasts/plasma cells were analyzed 120 h post stimulation by flow cytometry. Data are representative of three independent experiments yielding comparable results (**d**–**f**). *p *<* 0.05, **p *<* 0.01, ***p *<* 0.001 (unpaired *t* test). The source data are provided in Source Data file.

Butyrate and propionate can exert their activity by triggering cell signaling through G-protein-coupled receptors (GPRs), by functioning as energy substrate or by directly inhibiting HDACs, thereby modulating gene expression. None of the major SCFA receptors (Gpr43, Gpr41, Gpr109a, or OLFR78) were expressed on the surface of human resting or activated B cells; and only Gpr43 is expressed on activated mouse B cells (Supplementary Fig. 10a, b). This together with the failure of Gpr43 blocker GPLG0974 to interfere with butyrate- and propionate-mediated inhibition of CSR (Fig. 7a, b) demonstrated that SCFAs do not exert their activity on B cells by triggering cell signaling through GPRs. To address whether SCFAs function as energy substrate or HDAC inhibitors to modulate *Aicda* and *Prdm1* expression, CSR, and plasma cell differentiation, we stimulated mouse B cells with LPS plus IL-4 in the presence of nil, butyrate, propionate, long-chain fatty acid palmitate, or the non-SCFA HDAC inhibitors SAHA and TSA. Butyrate and propionate can function as an energy substrate by entering the tricarboxylic acid (TCA) cycle after breaking down to acetyl-CoA through β-oxidation. Palmitate can also function as an energy substrate^44^, after breaking down to acetyl-CoA, but cannot signal through GPR receptors or function as HDI. SAHA can only function as HDI, as it does neither enter the TCA cycle nor does it signal through GPRs. Consistent with the contention that butyrate and propionate inhibit *Aicda* and *Prdm1* expression through their HDAC inhibitory activity only, SAHA and TSA but not palmitate mimicked the activity of these SCFAs on *Aicda* and *Prdm1* expression, thereby reducing CSR and plasma cell differentiation (Fig. 7c–f; Supplementary Fig. 11a, b). Like butyrate and propionate, SAHA and TSA increased germline I_H_-C_H_ transcripts, and at low concentrations (SAHA, < 0.050 μM; TSA, < 0.0025 μΜ) moderately increased *Aicda* and CSR. Like butyrate and propionate, these non-SCFA HDAC inhibitors reduced *Aicda* and *Prdm1* expression, CSR, and plasma cell differentiation in a dose-dependent fashion.

Fluorometric analysis of HDAC activity in B cells stimulated with LPS plus IL-4 and in the presence of butyrate or propionate for 48 or 72 h confirmed the HDAC inhibitory activity of these SCFAs in B cells (Supplementary Fig. 8b), which led to increased acetylation of histone H3 in *Aicda* promoter, *Aicda* regulatory region 4, *Prdm1* promoter, as well as Iγ1, Iγ3, and Iα promoters (Supplementary Fig. 8c). The dispensability of butyrate and propionate as energy substrates was further emphasized by the lack of impact of etomoxir on the inhibition of CSR and plasma cell differentiation by these SCFAs (Supplementary Fig. 10c–e)—etomoxir prevents the formation of acylcarnitine, which is necessary for fatty acyl-chains transport from the mitochondria into the intermembrane space, thereby inhibiting fatty acid β-oxidation, which is required for butyrate-mediated energy metabolism and production of ATP^44^. Thus, SCFAs downregulate B cell *Aicda* and *Prdm1* through their HDAC inhibitory activity.

### SCFAs increase select miRNAs targeting *Aicda* or *Prdm1* 3′-UTR

**Fig. 8:**
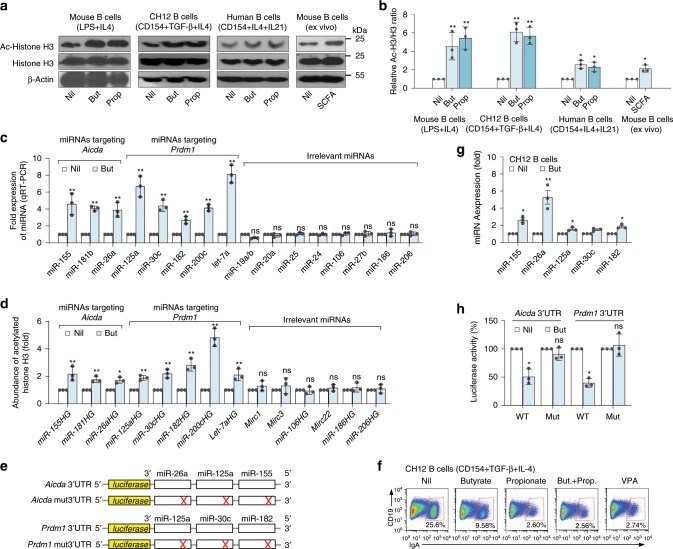
SCFAs increase expression of *Aicda-* and *Prdm1-*targeting miRNAs by enhancing histone acetylation of related miRNA host genes. **a** Primary mouse B cells, CH12F3 B cells, and human B cells were stimulated with indicated stimuli for 60 h in the presence of nil, butyrate (500 μM), or propionate (2000 μM). Ex vivo B cells were isolated from mice injected with NP-LPS and on plain water or SCFA water for 21 days. Acetylated-histone H3 (H3K9ac), histone H3, and β-actin proteins as detected by immunoblotting. Data are one representative of three independent experiments. **b** Relative densities of the Ac-histone H3 bands normalized to histone H3 and β*-*actin levels. **c**, **d** B cells stimulated with LPS plus IL-4 in the presence of nil or butyrate for 60 h. Expression of the *Aicda-* and *Prdm1-*targeting miRNAs, and irrelevant miRNAs as analyzed by qRT-PCR (**c**). Relative abundance of H3K9ac/K14ac in miRNA host genes (HGs) as analyzed by ChIP-qPCR (**d**). **e** Schematic diagram of the luciferase reporter constructs-containing 3′UTRs of *Aicda* and *Prdm1* mRNAs and their mutant (mut) counterparts. **f** Surface CD19 and IgA on CH12F3 B cells stimulated for 96 h in the presence of nil, butyrate (500 μM), propionate (2000 μM), or VPA (500 μM) as analyzed by flow cytometry. Data are one representative of 3 independent experiments yielding comparable results. **g**, miRNA expression in CH12F3 cells stimulated for 24 h in the presence of nil or butyrate, as analyzed by qRT-PCR. Data in **c**, **d**, and **g** are ratios to stimulated B cells cultured with nil (set as 1; means ± SEM of three independent experiments). **h** Luciferase activity in CH12F3 cells transfected with luciferase reporter vectors-containing wild-type or mutated *Aicda* or *Prdm1* 3′UTRs after 24 h treatment with nil or butyrate. Luciferase activity was measured 6 h after transfection, and normalized relative to luciferase activity in B cells cultured with nil. Data in **h** are ratios to transfected B cells cultured with nil (set as 100%; means ± SEM of three independent experiments). *p *<* 0.05, **p *<* 0.01, ns: not significant (unpaired *t* test). The source da*t*a are provided in Source Data file.

**Fig. 9:**
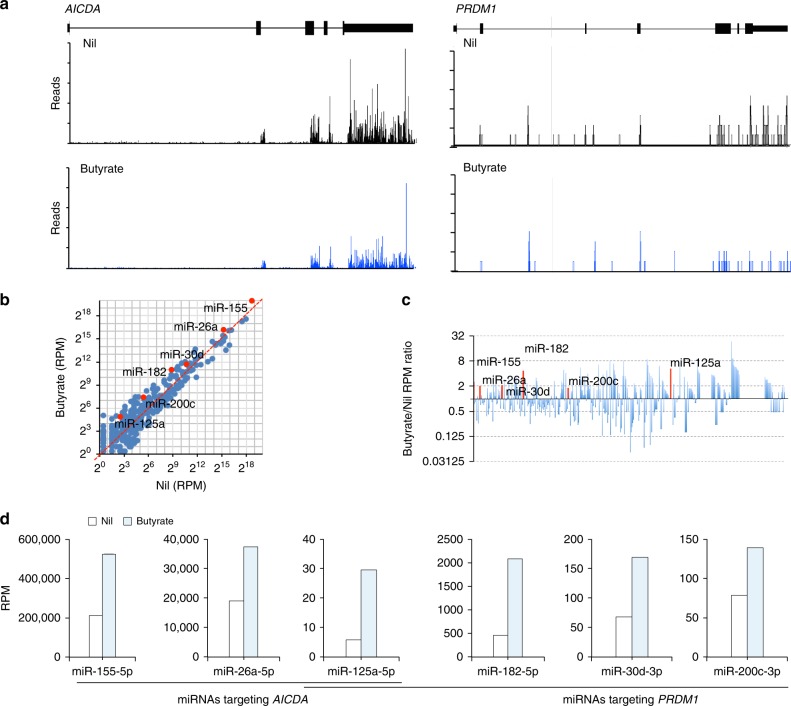
Butyrate downregulates *AICDA* and *PRDM1* expression while upregulating *AICDA-* and *PRDM1-*targeting miRNAs in human B cells. Purified human B cells were stimulated with CD154 plus IL-4 and IL-21 in the presence of nil or butyrate (500 μM) for 60 h before preparation of RNA for mRNA-Seq and miRNA-Seq. **a** Profile of RNA-Seq reads at the *AICDA* and *PRDM1* loci. **b** Scatter plots of miRNA expression as defined by miRNA-Seq (RPKs) in B cells treated with butyrate versus that in B cells treated with nil. **c** Bar graphs depict the fold changes in the average miRNA expression (RPMs) comparing B cells treated with nil or butyrate. **d** B-cell expression of *AICDA-* or *PRDM1-*targeting miRNAs, as analyzed by miRNA-Seq.

Acting as HDIs, butyrate and propionate increased overall histone (H3K9) acetylation in stimulated primary human and mouse B cells and CH12F3 B cells induced to undergo CSR and plasma cell differentiation, as well as and in B cells isolated from mouse on SCFAs water (Fig. 8a, b). As we have shown, HDIs, such as VPA, upregulate select miRNAs that target *Aicda* and *Prdm1* transcripts in B cells to silence the expression of these genes^8,33^. We hypothesized that SCFAs inhibit *Aicda* and *Prdm1* expression in a similar fashion. Indeed, butyrate selectively increased transcription of miR-155, miR-181b, and miR-26a, which target *Aicda* mRNA-3′UTR, concomitant with that of miR-30c, miR-182, miR-200c, and let-7a, which target *Prdm1* mRNA-3′UTR, and miR-125a, which targets both *Aicda* and *Prdm1* mRNA-3′UTRs, in mouse B cells (Fig. 8c; Supplementary Fig. 12). Increased transcription of these miRNAs reflected butyrate-mediated inhibition of the HDACs acting on the respective miRNA host genes (HGs), leading to hyperacetylation of miR155HG, miR-181bHG, miR-26aHG, and miR-125aHG (encoding miRNAs targeting *Aicda* 3′UTR) as well as miR30cHG, miR-182HG, miR-200cHG, and let-7aHG (encoding miRNAs targeting *Prdm1* 3′UTR) (Fig. 8d). The selectivity of butyrate in upregulating miRNAs that target *Aicda/AICDA* and *Prdm1/PRDM1* was emphasized by the failure of this SCFA to upregulate B-cell transcription of miR-19a/b, miR-20a, miR-25, miR27b, miR24, miR-106, miR-186, and miR-206, which are not known to regulate *Aicda or Prdm1* (Fig. 8c). In human B cells, butyrate selectively increased transcription of miR-155-5p, miR-26a-5p, and miR-125a-5p, which target *AICDA-*3′UTR, together with transcription of miR-182-5p, miR-30d-3p, miR-200c-3p, which target *PRDM1-*3′UTR (Fig. 9a–d).

To demonstrate that SCFAs-downregulation of AID and Blimp1 is mediated by upregulation of miRNAs targeting *Aicda* and *Prdm1* mRNA-3′ UTRs, we cloned wild-type and mutant (mut) sequences of *Aicda* and *Prdm1* 3′UTRs (mut 3′UTRs were constructed by mutation of the target sites of miR-26a, miR-125a, and miR-155 in *Aicda* mRNA-3′UTR or target sites of miR-125a, miR-30c, and miR-182 in *Prdm1* mRNA-3′UTR) into pMIR-REPORT luciferase reporter vectors (Fig. 8e). These were used to transfect mouse CH12F3 B cells, which were then induced to undergo CSR at a high rate. Like in primary B cells, butyrate upregulated miR-26a, miR-125a, miR-155, miR-125a, miR-30c, and miR-182 expression and reduced CSR in CH12F3 B cells stimulated with CD154 plus TGF-β and IL-4 (Fig. 8f, g). The ability of these butyrate-induced-miRNAs to repress luciferase activity was then analyzed using reporter constructs-containing wild-type or mutant *Aicda* and *Prdm1-*3′UTRs. Butyrate reduced the luciferase reporter activity in CH12F3 B cells transfected by wild-type, but not mutant reporter constructs (Fig. 8h). Thus, in B cells, butyrate inhibits histone deacetylation of the host genes of select miRNAs, which target *Aicda* and *Prdm1* mRNAs, thereby reducing AID and Blimp1 expression.

### SCFAs inhibit autoantibodies and autoimmunity in lupus-prone mice

**Fig. 10:**
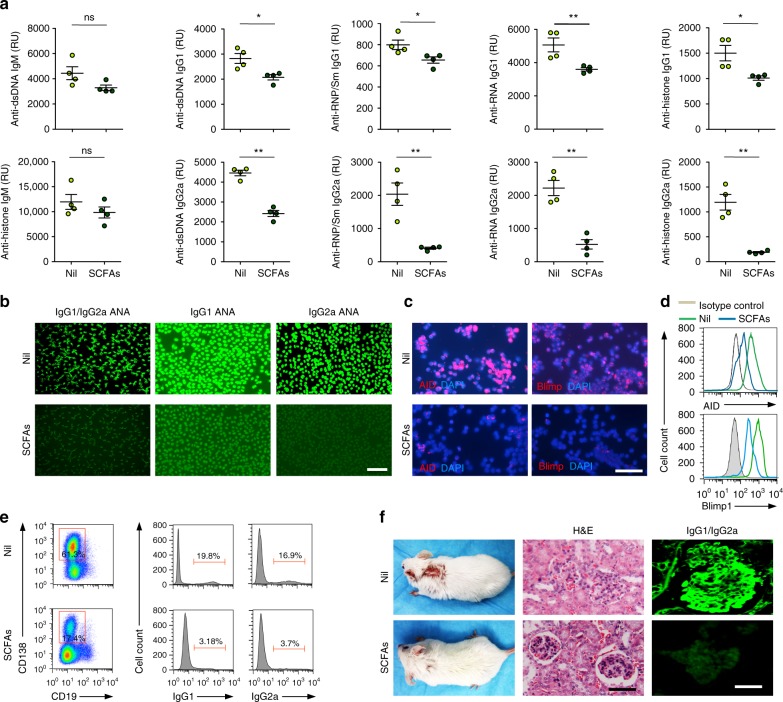
SCFAs reduce the autoantibody response and autoimmunity in lupus-prone mice. Female MRL/*Fas*^*lpr/lpr*^ mice fed fiber diet were given SCFAs water (SCFAs) or plain water (Nil) starting at the age of 5 weeks and killed at 17 weeks of age. **a** Titers of circulating anti-dsDNA IgM, and anti-dsDNA, anti-RNP/Sm, anti-histone, and anti-RNA IgG1 and IgG2a (relative units, RU), as analyzed by specific ELISAs. Each symbol represents an individual mouse (*n* = 4 per group, pooled from two experiments). The bars represent the mean ± SD. **b** ANA visualized by indirect immunofluorescence on HEp-2 cells that were incubated with sera (1:400 dilution) from the MRL/*Fas*^*lp/lpr*^ mice using FITC-labeled rat mAbs to mouse IgG1 and IgG2a. Data are from one of three independent experiments yielding comparable results. **c**, **d** AID and Blimp1 expression as analyzed by intracellular staining followed by fluorescence microscopy (**c**) and flow cytometry (**d**). **e** Spleen surface CD138^+^ plasmablasts/plasma cells, and intracellular CD138^+^ IgG1^+^ and CD138^+^ IgG2a^+^ plasmablasts/plasma cells as analyzed by flow cytometry. Data are representative of three independent experiments yielding comparable results. **f** Dorsal skin lesions (left panels), kidney sections stained with H&E (middle panels), and kidney sections stained with FITC-labeled rat mAbs to mouse IgG1 and IgG2a (right panels). Data are representative of three independent experiments. *p *<* 0.05, **p *<* 0.01, ***p *<* 0.001, ns: not significant (unpaired *t* test). Scale bar = 50 μm (**b**), 20 μm (**c**), and 100 μm (**f**). The source data are provided in Source Data file.

To address the impact of SCFAs on the autoantibody response, we fed four female lupus-prone MRL/*Fas*^*lpr/lpr*^ mice SCFAs water and four MRL/*Fas*^*lpr/lpr*^ mice plain water, starting at 5 weeks of age. We also fed five female lupus-prone NZB/W F1 mice SCFAs water and five NZB/W F1 mice plain water, starting at 14 weeks of age. These ages precede the initial clinical manifestations of lupus. At 12 or 26 weeks of age, respectively, MRL/*Fas*^*lpr/lpr*^ or NZB/WF1 mice drinking plain water displayed high titers of IgM autoantibodies to dsDNA and histone, IgG1 and IgG2a autoantibodies to dsDNA, RNP/Sm, RNA, histone, and nuclei, as well as increased B cell AID and Blimp1 expression (Fig. 10a–f; Supplementary Fig. 13a, b). They also developed IgG1^+^, IgG2a^+^, IgG2b^+^, and IgG3^+^B cells, CD138^+^IgG1^+^, CD138^+^IgG2a^+^, CD138^+^IgG2b^+^, and CD138^+^IgG3^+^ plasmablasts/plasma cells, skin lesions (MRL/*Fas*^*lpr/lpr*^), IgG1/IgG2 kidney deposition and glomerular sclerosis. By contrast, the MRL/*Fas*^*lpr/lpr*^ and NZB/W F1 mice drinking SCFAs water displayed significantly lower titers of IgG1 and IgG2a (not IgM) autoantibodies to dsDNA, RNP/Sm, anti-RNA, histone, and nuclei, as well as reduced B cell AID and Blimp1 expression (Fig. 10a–f; Supplementary Fig. 13c–f, 7). They also displayed reduced numbers of IgG1^+^, IgG2a^+^, IgG2b^+^, and IgG3^+^ B cells as well as CD138^+^IgG1^+^, CD138^+^IgG2a^+^, CD138^+^IgG2b^+^, and CD138^+^IgG3^+^ plasmablasts/plasma cells, and virtually no skin lesion. Finally, they showed no IgG1/IgG2a kidney deposition or glomerular damage. Thus, in lupus-prone mice, SCFAs dampen B cell AID and Blimp1 expression, plasma cell differentiation, systemic class-switched autoantibodies, and abolish lupus skin lesions and kidney pathology.

## Discussion

SCFAs derived from fermentation of resistant fibers by gut microbiota can impact functions of immune cells, including T cells, DCs, and macrophages^1,3,13,20,30,31^, and through these cells might impact antibody responses^1,4,9,10,20,27,40,45^. Here, we showed that butyrate and propionate act directly and, in a dose-dependent fashion, over a broad physiological range, on human and mouse B cells to downregulate AID and Blimp expression, thereby restricting CSR (to IgD, IgG, IgA, and IgE)/SHM and plasma cell differentiation through epigenetic modulation (expression of select miRNAs by histone acetylation of the related host genes) of *AICDA/Aicda* and *PRDM1/Prdm1* transcription (Supplementary Fig. 14). The significance of this SCFAs epigenetic impact on B cells was heightened by butyrate and propionate curtailing CSR/SHM and plasma cell differentiation in T-dependent and T-independent antibody responses to OVA in C57BL/6 mice, to NP in NP-CGG- and NP-LPS-injected C57BL/6 mice and NP-LPS-injected T-cell knockout (*Tcrβ*^*–/–*^*Tcrδ*^*–/–*^) mice, as well as in NP-LPS-injected NSG/B mice. The direct activity of SCFAs on B cells adds to the reported SCFA-mediated modulation of other immune cells, such as T cells, particularly Tregs, DCs, ILC2, ILC3, and macrophages^2,3,11,15,19,20,24,26–29,46^, and would critically contribute to maintaining a steady state balance between tolerance to commensal bacteria and immunity to pathogens, and, therefore, contributing to gut and systemic immune homeostasis.

Fermentation of dietary fibers by gut microbiota occurs primarily in distal jejunum, cecum, and proximal-medium colon. Butyrate is the preferred energy source for colonocytes and is consumed locally, before being resorbed, together with propionate and acetate throughout the colon, drained into the mesenteric veins and the portal vein to the liver, and, through the liver, to the rest of the body. Active transporters, such as the Na^+^-coupled SLC5a transporter and monocarboxylate transporter 1 (MCT1/SLC16a1), transport SCFA molecules across the cell membrane from an extracellular milieu of low SCFA concentration, such as in the circulatory torrent, into the cell, leading to higher intracellular SCFA concentrations. In this way, SCFAs accumulate in colon tissue, MLNs, and spleen at higher concentrations, higher enough for example to limit AID expression in inflamed colon mucosa, in which AID is induced by proinflammatory cytokines^47^. The SCFAs concentrations we used in vitro to inhibit AID and Blimp1 expression, CSR, and plasma cell differentiation reflected those in the spleen and MLNs of mice fed a regular chow (fiber diet), further emphasizing the physiological relevance of our findings. In our mice, butyrate and propionate, whether derived from gut fiber fermentation and/or SCFAs water, impacted B cells and antibody responses at both intestinal and systemic level. This resulting from systemic SCFAs impact on peripheral B cells and decreased input into the periphery of fewer class-switched AFCs, particularly IgA^+^ B cells, from the intestine due to high local SCFAs levels.

In addition to acting as HDIs after entering the cell via membrane diffusion or by monocarboxylate H^+^- or K^+^-coupled transporters, SCFAs can function as an energy substrate, to increase oxidative phosphorylation, glycolysis, and fatty acid synthesis. They can also modulate cell functions by binding to cell surface GPRs, such as GPR41, GPR43, GPR109A, or OLFR78, to trigger select signaling pathways, some of which with metabolic reach^1,9,48^. As we show here, *GPR41*, *GPR43*, *GPR109A*, and *OLFR78* genes are not expressed or virtually not expressed on human B cells, and only *Gpr43* is expressed on mouse B cells. Nevertheless, blocking B cell Gpr43 by GPLG0974 or culturing B cells with acetate, a GPR ligand functioning as an energy substrate but not possessing HDAC inhibitory activity, did not affect butyrate and propionate-mediated modulation of *Aicda* and *Prdm1*, CSR, or plasma cell differentiation. This together with the demonstration that etomoxir did not reverse the butyrate and propionate-mediated inhibition of the same processes, further argue against a role of SCFAs as energy substrate in promoting such B-cell processes. Thus, the notion suggested by a recent report^12^ that SCFAs boost IgG and IgA responses functioning as energy substrate is at odds with our exhaustive findings that butyrate and propionate display potent HDAC inhibitory activity in both human and mouse B cells, and directly modulate B-cell differentiation by acting as HDAC inhibitors, not as energy source or, for that matter, GPR signaling. This is further supported by the previous finding by us and others that, like butyrate and propionate, VPA, SAHA, TSA, and panobinostat (potent pharmacologic HDAC inhibitors that do not function as energy substrates or trigger GPR signaling in B cells) effectively dampened antibody or autoantibody responses^8,33,34,49^.

At low doses, butyrate and propionate increased expression of AID, germline I_H_-C_H_ transcripts and CSR in vitro, albeit at a low degree, a reflection of increased histone acetylation of *Aicda* regulatory regions and *IgH* locus, as mediated by SCFAs HDAC inhibitory activity. In mice immunized with NP-LPS, comparable low doses of SCFAs increased, at a low degree, AID and Blimp1 expression as well as the NP-binding IgG3 and IgG2b response. Our interpretation of these findings in vitro an in vivo is that while SCFA-induced *Aicda-* and *Prdm1-*targeting miRNAs inhibited *Aicda* and *Prdm1* in a dose-dependent fashion over a wide physiological (SCFAs) range, the *Aicda-* and *Prdm1-*targeting miRNAs-mediated inhibition of *Aicda* and *Prdm1* expression by low SCFAs doses was not robust enough to override the enhancement of CSR caused by the increased *Aicda* histone acetylation and germline I_H_-C_H_ transcription (Supplementary Fig. 14). This would also provide a mechanistic explanation for the moderate enhancement of class-switched antibodies reported by Kim et al.^12^.

Inhibition of *Aicda* and *Prdm1* expression by SCFAs was mediated by enhanced histone acetylation of host genes of select miRNAs targeting *Aicda* and *Prdm1* mRNAs. Like pharmacological HDI VPA^8,33^, butyrate enhanced the expression of miR-26a, miR-155, and miR-181b which target *Aicda*, and miR-30c, miR-182, miR-200c and let-7a which target *Prdm1*, as well as miR-125a which target both *Aicda* and *Prdm1* mRNAs, thereby implying a selectivity of these SCFAs for different HDACs^50^. Butyrate and, likely, propionate target and inhibit HDAC1, HDAC2, and HDAC3, and, to a lesser extent, other members of class I and class IIa HDACs^50,51^. Like histone acetyltransferases (HATs), HDACs do not directly bind to DNA; rather, they primarily associate with multiprotein complexes, the role and composition of which are often cell-type specific^52^. HDAC-associated proteins can further specify the selectivity of HDIs, which display different affinities for different HDAC/cofactor complexes^53^, as occurring in B lymphocytes, particularly when in an activated state. We refer to these compounded and sequential specificities of HDAC inhibitors as “HDAC inhibitors’ pathways of selectivity”. Finally, in addition to enhancing acetylation of histones, HDIs would increase acetylation of Drosha, an RNase III enzyme critical for miRNA biogenesis, and prevent its degradation by ubiquitination^54^. Thus, SCFAs not only enhance expression of host genes of select miRNAs targeting the *Aicda/AICDA* and *Prdm1/PRDM1* mRNA-3′UTRs but may also promote biogenesis of such miRNAs by increasing Drosha stability.

SCFAs-mediated modulation of antibody and autoantibody responses would be altered by changes of the gut microbiota. In human, the gut *Firmicutes*/*Bacteroidetes* ratio has been positively correlated with SCFAs concentration^55^. Accordingly, a higher *Firmicutes*/*Bacteroidetes* ratio in our mice fed a fiber diet was associated with a reduced class-switched antibody response, as compared with the mouse counterparts fed a no-fiber diet. Along the same line, a higher *Firmicutes/Bacteroidetes* ratio and an increased concentrations of SCFAs occur in obese people^56,57^, who display a deficient antibody response^58^, further supporting a critical role of SCFAs in modulating B-cell CSR/SHM and plasma cell differentiation. In one case, a low *Firmicutes*/*Bacteroidetes* ratio was similarly associated with increased IgA levels, but in mice on a high fiber diet^27^. This is seemingly at odd with our findings showing a strict relationship between fiber diet or high SCFAs concentrations and decreased IgA as well as IgG and IgE, and might have been influenced by other parameters, including mouse vendor (Jackson Laboratory vs. Harlan Laboratories)-dependent variations in gut microbiota and variations in husbandry facilities.

The gut microbiota elicits production of Ig of all isotypes, particularly IgA, as suggested by the greatly reduced IgA levels in germ-free animals^59^. Generally, most luminal IgAs are directed against intestinal flora^60,61^, possibly resulting from direct activation of B-cell BCR/TLRs by gut microbial commensals. Our demonstration of SCFA-mediated reduction of CSR to IgA in vitro as well as in vivo offers an explanation for the finding that administration of vancomycin, an antibiotic that clears a majority of SCFA-producing bacteria, leads to greatly increased serum IgA titers^60^. These may also result from increased levels of gut acetate, which enhances expression and/or activity of retinal dehydrogenase. In DCs, this converts vitamin A into its metabolite retinoic acid (RA), a booster of CSR to IgA^13,27^.

Our findings unveiled a direct and B-cell-intrinsic mechanism by which dietary fiber-derived SCFAs modulate B-cell differentiation processes that critically underpin effective T-dependent and T-independent antibody responses, as well as autoantibody responses. SCFAs-mediated decrease in autoantibody levels and amelioration of the skin and kidney immunopathology in lupus-prone MRL/*Fas*^*lpr/lpr*^ and NZB/W F1 mice open a possibility for a new potential therapeutic use of SCFAs in systemic lupus erythematosus. Like for CSR to IgG and IgA, SCFAs also inhibited CSR to IgE by acting directly on B cells and dampened total and antigen (OVA)-specific IgE titers, suggesting that SCFAs may also reduce IgE-mediated allergic response. In a physiological state, SCFAs would play an important role in maintaining host-defense homeostasis that prevents dysregulated antibody responses that can lead to autoimmunity or allergy. Further, they would keep the overall reactivity to digestive and respiratory commensals in a dynamic equilibrium. Such an equilibrium may be altered upon alterations of the gut microbiota (e.g., by bacterial or viral infections), leading to reduced levels of SCFAs and enhancement of antibody and autoantibody responses. Thus, our findings outline an important B-cell-intrinsic mechanism that takes cues from environmental epigenetic modifiers, i.e., dietary fibers and related catabolites by intestinal microbiota, to regulate B-cell differentiation processes critical to antibody and autoantibody responses.

## Methods

### Mice

C57BL/6, MRL/*Fas*^*lpr/lpr*^ (MRL/MpJ-*Fas*^*lpr*^/J, 000485), NZB/W F1 (NZB/WF1/J, 100008), *Tcrβ*^*–/–*^*Tcrδ*^*–/–*^ (B6129P2-*Tcrβ*^*tm1Mom*^*Tcrδ*^*tm1Mom*^/J, 002122)^42^, and NSG (NOD.Cg-*Prkdc*^*scid*^ *Il2rg*^*tm1Wjl*^/SzJ, 005557)^43^ mice were purchased from the Jackson Laboratory (Bar Harbor, Maine), *Aicda*^*−/−*^ mice (C57BL/6 background) were obtained from Dr. Tasuku Honjo (Kyoto University, Kyoto, Japan). The mice were housed and bred in our pathogen-free vivarium. All animal experiments were conducted in accordance with the guidelines and approved by the Institutional Animal Care and Use Committee (IACUC) of the University of Texas Health Science Center at San Antonio.

### Fiber diet, no-fiber diet, and SCFAs water

After weaning, mice were fed a regular chow diet, referred hereafter as “fiber diet” (Takland Mouse/Rat Sterilizable Diet LM-485—containing 18% fibers) or a no-fiber diet (Harlan laboratories TD.00129 AIN-93G No Cellulose—containing 0% fibers). Mice fed a fiber or no-fiber diet were then given drinking water-containing SCFAs (SCFAs water, pH = 7.4, containing 20.0 mg ml^−1^ tributyrin emulsion, 140 mM sodium butyrate, and 150 mM sodium propionate), water containing a low dose of SCFAs (pH = 7.4, 20 mM sodium butyrate, and 30 mM sodium butyrate) or plain water (pH = 7.4, sodium-adjusted to match SCFAs water) starting at the age of 5 weeks. Tributyrin is a triglyceride butyrate analog which has a long in vivo half-life and act as a pro-drug of butyrate. It is cleaved by intracellular lipases and hydrolyzed into three molecules of butyrate^62^. In mice fed the fiber diet, administration of the SCFAs water resulted in 20–200 μM, or 18–20 μmol g^–1^ butyrate and propionate, in the circulation and feces, respectively. Drinking water-containing SCFAs at the above concentration was always well accepted by all mice used in this study.

### SCFAs modulation of T-dependent and T-independent antibody responses

To analyze local (intestinal) and systemic T-dependent antibody response to OVA, C57BL/6 mice fed a fiber or no-fiber diet, and SCFAs water or plain water ad libitum, were administered OVA (20 mg) plus mucosal adjuvant cholera toxin (List Biological Labs, 20 μg) in 500 μl PBS via intragastric gavage once a week for 4 weeks, and killed 1 week after the last OVA and cholera toxin administration for all studies. For analysis of the T-dependent antibody response to NP, C7BL/6 mice were injected i.p. at 8 weeks of age with 100 μg of NP_16_-CGG (Biosearch Technologies) in 100 μl of alum (Inject Alum, Pierce) and boost-injected with 100 μg of NP_16_-CGG in PBS 3 weeks later. For analysis of the T-independent antibody response to NP, C7BL/6 or *Tcrβ*^*–/–*^*Tcrδ*^*–/–*^ mice were fed a fiber diet and SCFAs water or plain water ad libitum, and injected i.p. at 8 weeks of age with 50 μg of NP-LPS (Biosearch Technologies) in 100 μl of PBS, and boost-injected with 50 μg of NP-LPS in 100 μl of PBS 3 weeks later. All mice were killed 1 week after the boost injection.

### NSG/B mice

NSG/B mice were generated by grafting NSG mice with highly purified C57BL/6 B cells. Such B cells were purified from splenocytes of 8–12 weeks old C57BL/6 mice by negative selection using the EasySep™ Mouse B Cell Isolation Kit (STEMCELL Technologies) following the manufacturer’s instructions. Purified B cells were injected intravenously (20.0 × 10^6^ cells per mouse in 250 μl PBS in lateral tail veins) into 8-week-old NSG mice. After B-cell engraftment, mice were stated on SCFAs water or plain water (until the end of the experiment) and i.p. injected with NP-LPS (50 μg in PBS) at days 0, 2, 4, and 6, and killed 1 week after the last NP-LPS injection.

### Detection of antibodies and autoantibodies

Titers of IgM, IgD, IgG, IgG1, IgE and IgA from in vitro culture supernatants of stimulated human and mouse B cells or titers of circulating or fecal total and/or NP-binding IgM, IgG1, IgA, IgG2b, and IgG3 were measured using specific ELISAs, as we described^33,63–65^. OVA-binding IgE were detected by sandwich ELISAs, using plates coated with rat anti-mouse IgE mAb (23G3, Southern Biotech) (Supplementary Table 1). Serial twofold diluted serum samples in PBS-(0.05%) Tween 20 (PBS-Tween 20) were added to the plates and incubated for 2 h at 23 °C. After washing with PBS-Tween 20, biotin-OVA (152060, GALAB) was added. After a final washing, IgE-bound antigens were detected using streptavidin-horseradish peroxidase.

Anti-dsDNA, anti-RNP/Sm, anti-histone, and anti-RNA IgM, IgG1, and IgG2a autoantibody titers in sera of MRL/*Fas*^*lpr/lpr*^ and NZB/W F1 mice were measured by specific ELISAs, as described^33,66^, except that for anti-RNP/Sm, anti-histone, and anti-RNA Abs, plates were coated with RNP/Sm (MBS318132, MyBioSource), histone (H9250, Sigma-Aldrich) or mouse liver total RNA, respectively. Antigen-specific antibody titers are expressed as relative units (RU), defined as the dilution factor needed to reach 50% saturation binding, as calculated using GraphPad Prism^®^ software (GraphPad). To detect antinuclear antibody (ANA), sera were serially diluted in PBS (from 1:50 to 1:400), incubated on antinuclear Ab substrate slides (HEp-2 cell-coated slides, MBL-BION), and detected with FITC–anti-IgG1 (A85–1, BD Biosciences), FITC–anti-IgG2a mAb (R19-15, BD Biosciences), or 1:1 mixture of FITC–anti-IgG1 and FITC–anti-IgG2a mAbs. Images were acquired with a ×20 objective on a fluorescence microscope.

To analyze kidney IgG deposition, kidneys from MRL/*Fas*^*lpr/lpr*^ and NZB/W F1 mice were fixed in paraformaldehyde (PFA, 4%) and paraffin embedded for immunofluorescence imaging. For immunofluorescence, 5 μm sections were prepared by cryostat, loaded onto positively charged slides, fixed in cold acetone, and stained with a mixture of anti- to mouse IgG1 FITC-labeled rat mAb and anti- to mouse IgG2a FITC-labeled rat mAb. Cover slips were mounted using ProLong^®^ Gold Antifade Reagent, before examination under the fluorescence microscope.

### Detection of bacteria-binding and bacteria-bound antibodies

Serum bacteria-binding IgM and IgA were analyzed by ELISA using plates coated with bacteria. Freshly cultured *E. coli* were washed with PBS, heat inactivated at 65 °C for 15 min, and resuspended in water (200 μg ml^−1^). In total, 50 μl of bacteria suspension was added to each well of a 96-well ELISA plate and dried overnight in a 37 °C incubator. Plates were washed three times with PBS-Tween 20 and blocked with 1% (v/w) BSA in PBS for 1 h at room temperature. Mouse sera were serially twofold diluted from 1:20 and incubated for 2 h at 37 °C. Bacteria-binding IgM and IgA were then detected using anti-mouse IgM or anti-mouse IgA mAb.

To detect bacteria-bound IgM, IgD, IgG1, and IgA, feces (10 mg) were suspended in 100 μl 1x PBS (filtered through 0.2 -μm filter), homogenized and centrifuged at 400×*g* for 5 min to remove large particles. The supernatant was then centrifuged at 8000×*g* for 10 min to remove non-bound antibodies (in supernatant). The bacterial pellet was suspended in 1 ml of PBS with 1% (w/v) BSA. After fixation with 7.2% formaldehyde for 10 min at room temperature, bacteria were washed with PBS, and stained with PE–anti-IgM mAb (AF6-78, BD Biosciences), FITC–anti-IgG1 mAb (406606, BD Biosciences) or FITC–anti-IgA mAb (C10-3, BD Biosciences) on ice for 30 min, washed with PBS, and further resuspended in 1× PBS containing 0.2 μg ml^−1^ DAPI for flow cytometry analysis. All events that stained with DAPI were considered as bacteria.

### SCFAs concentrations and HDAC activity

Concentrations of SCFAs in the spleen, colon tissues, and MLNs were analyzed by a Gas chromatography–mass spectrometry (GC-MS) method (Creative Proteomics). The tissues were homogenized in 1.0 ml of ethanol (containing 0.5% HCl, V/V) by ultrasonication. The homogenates were centrifuged at 12,000 *g* for 10 min. The supernatants were analyzed using a GC-MS Agilent 7890-5977 system. μM concentrations of tissue SCFAs were derived from μmol g^−1^ tissue concentrations using the formula: μM (μmol per l) = μmol Kg^−1^ divided by tissue density (g ml^−1^). Fecal and serum SCFA concentrations were analyzed by the UT Health Long School of Medicine Mass Spectrometry Laboratory using a LC-MS/MS system (Q Exactive™ Hybrid Quadrupole-Orbitrap Mass Spectrometer, Thermo Fisher). Feces (100 mg) were first homogenized in 400 μl of deionized water by crushing and vortex them vigorously until all solid content passed into solution. Acidify sera and fecal samples were added with 25% metaphosphoric acid at 1:5 ratio (one volume of acid for five volumes of sample) for 30 mins on ice. Samples were then centrifuged at 12,000 *g* for 15 min at 4 °C, and supernatants were filtered using Ultrafree MC columns (Millipore). The HDAC inhibitory activity of SCAFs was analyzed in B cells stimulated with LPS plus IL-4 in the presence of nil or different concentrations of sodium acetate, sodium butyrate, or sodium propionate for 48 or 72 h. In such B cells, the HDAC activity was measured using a HDAC Cell-Based Activity Assay kit (600150, Cayman).

### Microbiota analysis

Microbial DNA was extracted from mouse feces using Quick-DNA™ Fecal/Soil Microbe Microprep Kit (Zymo Research) according to the manufacturer’s instructions. To determine the composition of the bacterial phyla present in feces of the mice fed different diets, isolated bacterial DNA was tagged and sequenced using miSeq platform. The V3-V4 hypervariable region of the bacteria 16S rRNA gene was amplified by PCR using tagged bact-341F primer 5′-TCGTCGGCAGCGTCAGATGTGTATAAGAGACAGCCTACGGGNGGC WGCAG-3′ and bact-805R primer 5′-GTCTCGTGGGCTCGGAGATGTGTATAAGAGACAGGACTACHVGGGTATCTAATCC-3′ and Phusion DNA polymerase. Multiplexing indices and Illumina sequencing adapters were then added to the amplicons by limited-cycle amplification using the Nextera XT Index Kit (Illumina). The libraries were normalized and pooled, and sequenced on the MiSeq system (Illumina). Sequencing and quality assessment were performed at UT Health Genome Sequencing Facility. Bacterial taxonomy was assigned using the Ribosomal Database Project (RDP) classifier^67^.

### Detection of antibody-forming cells (AFCs)

Spleen, bone marrow, MLN, and PP cells were isolated from desired mice and analyzed for AFCs by ELISPOT. MultiScreen^®^ ELISPOT plates (MAIPS4510, Millipore) were activated with ethanol (35%), washed four times with PBS and coated with unlabeled rabbit polyclonal antibodies against mouse IgM, IgG1, or IgA in PBS overnight at 4 °C. The plates were then washed six times with PBS, blocked with BSA (0.5%) in RPMI/HEPES plus L-glutamine for 1 h at room temperature. Single-cell suspensions from lamina propria, bone marrow, and spleen cells of OVA-immunized mice were cultured in the plates at 37 °C for 16 h in FCS-RPMI at 250,000, 125,000, and 75,000 cells per well. The cultures were then removed, the plates were washed six times, incubated with biotin-anti-IgM, IgG1, or IgA for 2 h on a shaker at room temperature, washed, incubated with horseradish peroxidase (HRP)-streptavidin (Santa Cruz Biotech) for 1 h on a shaker at room temperature, washed again and developed using the Vectastain AEC peroxidase substrate kit following the manufacturer’s protocol (SK-4200, Vector Laboratories). Plates were imaged and quantified using a CTL-ImmunoSpot^®^ Analyzer and software.

### Flow cytometry

For surface staining, mononuclear cells were reacted with VF-anti-CD19 mAb (75-0193-0100, Tonbo), PE–anti-IgM mAb (406507, BioLegend), APC-anti-IgG1 mAb (406609, BioLegend), FITC–anti-IgA mAb (C10-3, BD Biosciences), 7-AAD, PE-Cy7-anti-CD138 mAb (142513, BioLegend), APC-anti-CD3 mAb (100221, BioLegend), APC-anti-CD4 (GK1.5, BioLegend), and/or APC-anti-CD8 mAb (53-6.7, BD Biosciences). For intracellular staining, cells were stained with anti-CD19 mAb (Clone 1D3; Tonbo) and fixable viability dye eFluor^®^ 450 (FVD 450, eBiosciences) followed by incubation with the BD Cytofix/Cytoperm buffer at 4 °C for 20 min. After washing twice with the BD Perm/Wash buffer, cells were resuspended in HBSS with 1% BSA and stored overnight at 4 °C. Cells were then stained with FITC–anti-AID Ab (bs-7855R-FITC, Bioss), APC-anti-Blimp1 mAb (5E7, BioLegend), PE-Cy7-anti-CD138 mAb, APC-anti-IgG1 mAb (406610, BioLegend), FITC–anti-IgG2a mAbs (553390, BD Biosciences), and/or PE-anti-IgE mAb (23G3, eBioscience). FACS analysis was performed on single-cell suspensions. In all flow cytometry experiments, cells were appropriately gated on forward and side scattering to exclude dead cells and debris. Cell analyses were performed using a LSR-II flow cytometer (BD Biosciences), and data were analyzed using FlowJo software (TreeStar). All experiments were performed in triplicates.

### Fluorescence microscopy

Fluorescence microscopy of tissues. To analyze IgM, IgD, and IgA-producing cells in the lamina propria and PPs, the intestine was folded into a “Swiss-roll”, fixed with PFA (4%), and embedded in paraffin. Ten micrometer sections were cut and heated at 80 °C to adhere to the slide, washed four times in xylene for 2 min, dehydrated two times with 100% ethanol for 1 min, two times with 95% ethanol for 1 min, and washed two times in water for 1 min. Antigens were unmasked using 2 mM EDTA in 100 °C for 40 mins followed by a cooling step at 25 °C on the bench top, three times washing with 1x TBS and blocking using 10% BSA for 15 min. Slides were again washed three times with 1x TBS and stained with PE–anti-IgM, primary-rabbit anti-mouse-IgA mAb (PA-1-30826, Thermo Fisher) followed by Alexa Fluor 488^®^-anti rabbit-IgG (H + L) F(ab’)_2_ mAb (4412, Cell Signaling), and FITC–anti-IgD mAb (405713, BioLegend), PE goat-anti-mouse-IgM mAb (406507, BioLegend) for 2 h in a moist dark chamber. After washing three times with Triton X-100 (0.1%) in TBS, slides were air dried, and cover slips were mounted with ProLong^®^ Gold Antifade Reagent with DAPI (Invitrogen). Fluorescence images were captured using a 10x objective lens with a Zeiss Axio Imager Z1 fluorescence microscope. To analyze the germinal center structure in spleen or IgD-producing cells in MLNs, 10 μm spleen or MLN sections were prepared by cryostat and loaded onto positively charged slides, fixed in cold acetone and stained with PE–anti-GL-7 (144607, BioLegend) and FITC–anti-B220 mAbs (103206, BioLegend), or FITC–anti-IgD mAb (405704, BioLegend), respectively, for 1 h at 25 °C in a moist chamber. Cover slips were then mounted using ProLong^®^ Gold Antifade Reagent, before examination with a fluorescence microscope.

Fluorescence microscopy of B cells. B cells were suspended at 0.1 × 10^6^ cells per100 μl in FCS-RPMI. Pre-labeled slides were then placed into cytofunnels and ran with 50 μl of FCS-RPMI, in order to wet the cytofunnel paper. Cells were then placed again into the cytofunnel and spun at 800 RPM for 3 mins using Cytospin™ 4 Cytocentrifuge (Thermo Fisher). For intracellular visualization of AID and Blimp1 proteins, the cells were fixed with methanol for 15 min and washed three times in PBS-Tween 20. Cells were then blocked in 10% BSA for 15 min and stained with 1:20 Alexa 647-AID (bs-7855R-A647, Bioss), or Alexa 647-Blimp1 (150003, BioLegend) overnight in a dark moist chamber.

### Human and mouse B cells, CSR, and plasma cell differentiation

Mouse IgD^+^ naïve B cells were isolated from 8- to 12 weeks-old C57BL/6 mice as described^33,64,65^. B cells were resuspended in FCS-RPMI containing 50 mM β-mercaptoethanol and 1x antibiotic-anti-mycotic mixture (15240-062, Invitrogen) at 37 °C in 48-well plates and stimulated with LPS (3 μg ml^−1^) or CD154 (1 U ml^−1^, obtained from membrane fragments of CD154 encoding recombinant baculovirus-infected Sf21 insect cells) plus IL-4 (5 ng ml^−1^, R&D Systems) for CSR to IgG1, IgE and plasma cell differentiation, LPS (3 μg ml^−1^) or CD154 (1 U ml^−1^) plus TGF-β (2 ng ml^−1^, R&D Systems), IL-4 (5 ng ml^−1^), IL-5 (3 ng ml^−1^, R&D Systems), anti-δ mAb-dextran (Fina Biosolutions) and RA (10 nM) for CSR to IgA, or LPS (3 μg ml^−1^) plus TGF-β (2 ng ml^−1^), IL-4 (5 ng ml^−1^), IL-5 (3 ng ml^−1^) and RA (10 nM) for CSR to IgD. Butyrate (50–500 μM), propionate (25–2000 μM), palmitate (64–125 μM), (R)-( + )-etomoxir (25 μM), SAHA (0.2 or 0.3 μM), TSA (0.001–1.6 μM), or GLPG0974 (0.1–5.0 μM) were added to cultures, and cells were collected at various times.

Human IgD^+^ naïve B cells (~99% pure) were purified by negative selection from healthy donor PBMCs using the EasySep Human Naive B Cell Enrichment Kit (19254, STEMCELL Technologies), following the manufacturer’s instructions. Naive B cells were then cultured in FCS-RPMI and stimulated with CD154 (10 U ml^−1^), IL-4 (20 ng ml^−1^, R&D Systems), and IL-21 (50 ng ml^−1^, R&D Systems) or CD154 (10 U ml^−1^), IL-21 (50 ng ml^−1^), and TGF-β (0.5 ng ml^−1^) in the presence of nil, butyrate, propionate, or butyrate plus propionate for 120 h. B cells were then stained with 7-aminoactinomycin D (7-AAD), FITC–anti-IgM mAb (314506, BioLegend), PE–anti-CD19 mAb (302208, BioLegend), and allophycocyanin-anti-IgG mAb (562025, BD Biosciences) or 7-AAD, FITC–anti-IgA mAb (F5259, Sigma-Aldrich), and biotin-F(ab′)_2_ anti-IgM (2022-08, Southern Biotech), followed by allophycocyanin–streptavidin, and analyzed by flow cytometry.

### Immunoblotting

B cells from C57BL/6 mice were stimulated with LPS (3 μg ml^−1^) plus IL-4 (4 ng ml^−1^) in the presence of nil, butyrate (500 μM), propionate (2000 μM), or butyrate (500 μM) plus propionate (2000 μM) for 72 h. Cells were harvested and lysed in Laemmli buffer. Cell extracts containing equal amounts of protein (20 μg) were fractionated through SDS-PAGE (10%). The fractionated proteins were transferred onto polyvinylidene difluoride membranes (Bio-Rad Laboratories) overnight (30 V) at 4 °C. After blocking and overnight incubation at 4 °C with anti-AID (ZA001, Invitrogen), anti-Blimp1 (6D3, eBioscience), anti-acetyl-histone H3 (H3K9ac/K14ac, 17–615, Millipore), anti-histone H3 (601901, BioLegend), or anti-β-Actin mAb (AC-15, Sigma-Aldrich), the membranes were incubated with HRP-conjugated secondary Abs. After washing with PBS-Tween 20, bound HRP-conjugated Abs were detected using Western Lightning^®^ Plus-Enhanced Chemiluminescence reagents (PerkinElmer Life and Analytical Sciences).

### Quantitative RT-PCR of mRNA, miRNA, germline, post-recombination, and mature transcripts

For quantification of mRNA, pri-miRNA, germline I_H_-C_H_, post-recombination Iμ-C_H_, and mature V_H_DJ_H_-C_H_ transcripts, RNA was extracted from 0.2–5.0 × 10^6^ cells using either Trizol^®^ Reagent (Invitrogen) or RNeasy Plus Mini Kit (Qiagen). Residual DNA was removed from the extracted RNA with gDNA eliminator columns (Qiagen). cDNA was synthesized from the total RNA with the SuperScript^TM^ III First-Strand Synthesis System (Invitrogen) using oligo-dT primer. Transcript expression was measured by qRT-PCR with the appropriate primers (Supplementary Table 2) using a Bio-Rad MyiQ^TM^ Real-Time PCR Detection System (Bio-Rad Laboratories) to measure SYBR Green (IQ^TM^ SYBR^®^ Green Supermix, Bio-Rad Laboratories) incorporation with the following protocol: 95 °C for 15 s, 40 cycles of 94 °C for 10 s, 60 °C for 30 s, 72 °C for 30 s. Data acquisition was performed during 72 °C extension step. Melting curve analysis was performed from 72 to 95 °C. For quantification of mature miRNA transcripts, RNA was extracted from 0.2–5.0 × 10^6^ cells using miRNeasy^®^ Mini Kit (Qiagen) and then reverse-transcribed with miScript II RT Kit (Qiagen) using the miScript HiSpec buffer. A Bio-Rad MyiQ^TM^ Real-Time PCR Detection System was used to measure SYBR Green (miScript SYBR Green PCR Kit, Qiagen) incorporation according to the manufacturer’s instructions. Mature miRNA forward primers (Supplementary Table 1) were used at 250 nM in conjunction with the Qiagen miScript Universal Primer and normalized to expression of small nuclear/nucleolar RNAs Rnu6/RNU61/2, Snord61/SNORD61, Snord68/SNORD68, and Snord70/SNORD70. The ΔΔCt method was used for data analysis of qRT-PCR experiments.

### SHM analysis

To analyze Ig SHM induced in response to NP immunization, spleen B cells were isolated from NP_16_-CGG-injected C57BL/6 mice, NP-LPS-injected C57BL/6, *Tcrβ*^*–/–*^*Tcrδ*^*–/–*^, and NSG/B mice for RNA extraction using the RNeasy Mini Kit (Qiagen). cDNA was synthesized from 1 to 2 μg of the total RNA with the SuperScript™ III First-Strand Synthesis System (Invitrogen) using the oligo-dT primer. Rearranged V_186.2_DJ_H_-Cγ1, V_186.2_DJ_H_-Cγ2b, or V_186.2_DJ_H_-Cγ3 cDNA encoding the anti-NP IgH chain was amplified using a V_186.2_ leader-specific forward primer together with a reverse Cγ1-, Cγ2b-, or Cγ3-specific primer^33^ tagged with Illumina clustering adapters and Phusion™ high-fidelity DNA polymerase (New England BioLabs). Amplification conditions were 98 °C for 10 s, 60 °C for 45 s, and 72 °C for 1 min for 30 cycles. The amplified library was tagged with barcodes for sample multiplexing, PCR enriched, and annealed to the required Illumina clustering adapters. High-throughput 300 bp pair-ended sequencing was performed using the Illumina MiSeq system. Somatic mutations in V_186.2_ (IMGT V1-72) segments were analyzed using IMGT*/*HighV-QUEST (The international ImMunoGeneTics information system^®^, http://www.imgt.org).

### Analysis of histone acetylation by ChIP-qPCR

ChIP assays were performed as described^6,33,64,65^. B cells (1 × 10^7^) were treated with formaldehyde (1% v/v) for 10 min at 25 °C to cross-link chromatin, washed once in cold PBS with protease inhibitors (Roche), and resuspended in lysis buffer (20 mM Tris-HCl, 200 mM NaCl, 2 mM EDTA, 0.1% w/v SDS, and protease inhibitors, pH 8.0). Chromatin was sonicated to yield DNA fragments (about 200 to 1000 bp in length), pre-cleared with protein A agarose beads (Pierce) and incubated with anti-acetyl-histone H3 mAb (H3K9ac/K14ac, 17–615, Millipore) at 4 °C overnight. Immune complexes were precipitated by Protein A agarose beads, washed and eluted (50 mM Tris-HCl, 0.5% SDS, 200 mM NaCl, 100 μg ml^−1^ proteinase K, pH 8.0), followed by incubation at 65 °C for 4 h. DNA was purified using a QIAquick PCR purification kit (Qiagen). The miRNA host gene,* Aicda*, *Prdm1* and I_H_ promoter region DNAs were amplified from immunoprecipitated chromatin by qPCR using appropriate primers (Supplementary Table 1). Data were normalized to input chromatin DNA and depicted as relative abundance of each amplicon.

### High-throughput mRNA-Seq and miRNA-Seq

The total RNA was extracted from 2 × 10^6^ cells using miRNeasy^®^ Mini Kit (Qiagen), as described^33^. RNA integrity was verified using an Agilent Bioanalyzer 2100 (Agilent). Next-generation mRNA-Seq and small RNA-Seq were performed by the UT Health Genome Sequencing Facility^8^. High-quality RNA (RNA integrity number or RIN.9.0) was processed using an Illumina TruSeq RNA sample prep kit v2 or TruSeq Small RNA Sample Prep kit following the manufacturer’s instructions (Illumina). Clusters were generated using TruSeq Single-Read Cluster Gen. Kit v3-cBot-HS on an Illumina cBot Cluster Generation Station. After quality control procedures, individual mRNA-Seq or small RNA-Seq libraries were then pooled based on their respective 6-bp index portion of the TruSeq adapters and sequenced at 50 bp/sequence, read using an Illumina HiSeq 3000 sequencer. The barcode combinations were further crosschecked by Illumina Experiment Manager software. Sequence data were checked by assurance (QA) pipeline and initial genome alignment (Alignment). Approximately, 33 million and 5 million reads per sample were generated in mRNA-Seq and miRNA-Seq, respectively. After the sequencing run, demultiplexing with CASAVA was employed to generate the Fastq file for each sample. All sequencing reads were aligned with their reference genome (UCSC mouse genome build mm9) using TopHat2 default settings, and the Bam files from alignment were processed using HTSeq-count to obtain the counts per gene in all samples. Quality control statistical analysis of outliers, intergroup variability, distribution levels, PCA, and hierarchical clustering analysis were performed for statistical validation of the experimental data.

### Luciferase reporter assay

The 3′UTRs of *Aicda* and *Prdm1* mRNAs were PCR amplified using Phusion DNA polymerase (New England BioLabs.) from cDNA of B cells stimulated with LPS plus IL-4 and cloned into the pMIR-REPORT miRNA Expression Reporter Vector System (Invitrogen), which allows for analysis of 3′UTR-mediated regulation of firefly luciferase activity. The mutant (mut) *Aicda* 3′UTR containing point mutations in the seed sequences of miR-26a, miR-125a, and miR-155 target sites, and the mut *Prdm1* 3′UTR containing point mutations in the seed sequences of miR-30c, miR-125a, and miR-182 target sites were generated by PCR-based mutagenesis of the *Aicda* 3′UTR pMIR-REPORT, or the *Prdm1* 3′UTR pMIR-REPORT vector, respectively^33^. The sequence of constructs was confirmed by two independent sequencing reactions. Reporter constructs were co-transfected with the pRL-TK vector (Promega), which drives constitutive expression of *Renilla reniformis* luciferase, into mouse CH12F3 B cells (a gift from Dr. Tasuku Honjo) by electroporation (250 V and 900 Ω) with a Gene Pulser II (Bio-Rad). Transfected CH12F3 B cells were then stimulated with CD154 (1 U ml^−1^), IL-4 (5 ng ml^−1^), and TGF-β (2 ng ml^−1^) to induce AID expression and CSR to IgA) in the presence nil or butyrate (500 μM). The ability of butyrate to repress reporter activity was determined by firefly luciferase activity and normalized to *Renilla* luciferase activity, according to the manufacturer’s instructions, using the Luc-Pair™ Duo-Luciferase HS Assay Kit (GeneCopoeia).

### Statistical analyses

All statistical analyses were performed using Excel (Microsoft) or GraphPad Prism^®^ software. Differences in Ig titers, CSR, and RNA transcript expression were analyzed with Student’s paired (in vitro) and unpaired (in vivo) *t* test assuming two-tailed distributions. Differences in the frequency and spectrum of somatic point mutations were analyzed with χ^2^ tests. A *p*-value of < 0.05 was considered significant.

### Reporting summary

Further information on research design is available in the Nature Research Reporting Summary linked to this article.

## Supplementary information

Supplementary Info

Reporting Summary

## Data Availability

The mRNA-sequencing and microRNA-sequencing data have been deposited in NCBI Gene Expression Omnibus (GEO) and is accessible through GEO Series accession number GSE140111. VDJ-sequencing and 16S rRNA gene-sequencing data have been deposited in NCBI Sequence Read Archive (SRA) under BioProject PRJNA588067. The source data underlying Figs. 1a, b, 2c, f, g, 3a–h, 4a, d, g, i, 5h–j, l, 6c, d, 7a, c, 8a–d, g, h, 9d and 10a and Supplementary Figs. 1a, b, 4a–d, 5, 6c, d, 8a–c, 10a, b, 11a, and 13a are provided as a Source Data file. All the other data supporting the findings of this study are available within the article and its Supplementary Information Files and from the corresponding author upon reasonable request.

## Source data

Source Data
