# Supplementary material for: B cell-intrinsic epigenetic modulation of antibody responses by dietary fiber-derived short-chain fatty acids
Source: Nat Commun. 2020 Jan 2;11:60. doi: 10.1038/s41467-019-13603-6 (PMC6940392; doi:10.1038/s41467-019-13603-6)
Supplement: Supplementary file 1 — Supplementary Info [file 41467_2019_13603_MOESM1_ESM.pdf]

Supplementary Information

B cell-intrinsic epigenetic modulation of the antibody response by dietary fiber-derived short-chain fatty acids

Helia N. Sanchez, Justin B. Moroney, Huoqun Gan, Tian Shen, John L. Im, Tianbao Li, Julia R. Taylor, Hong Zan and Paolo Casali

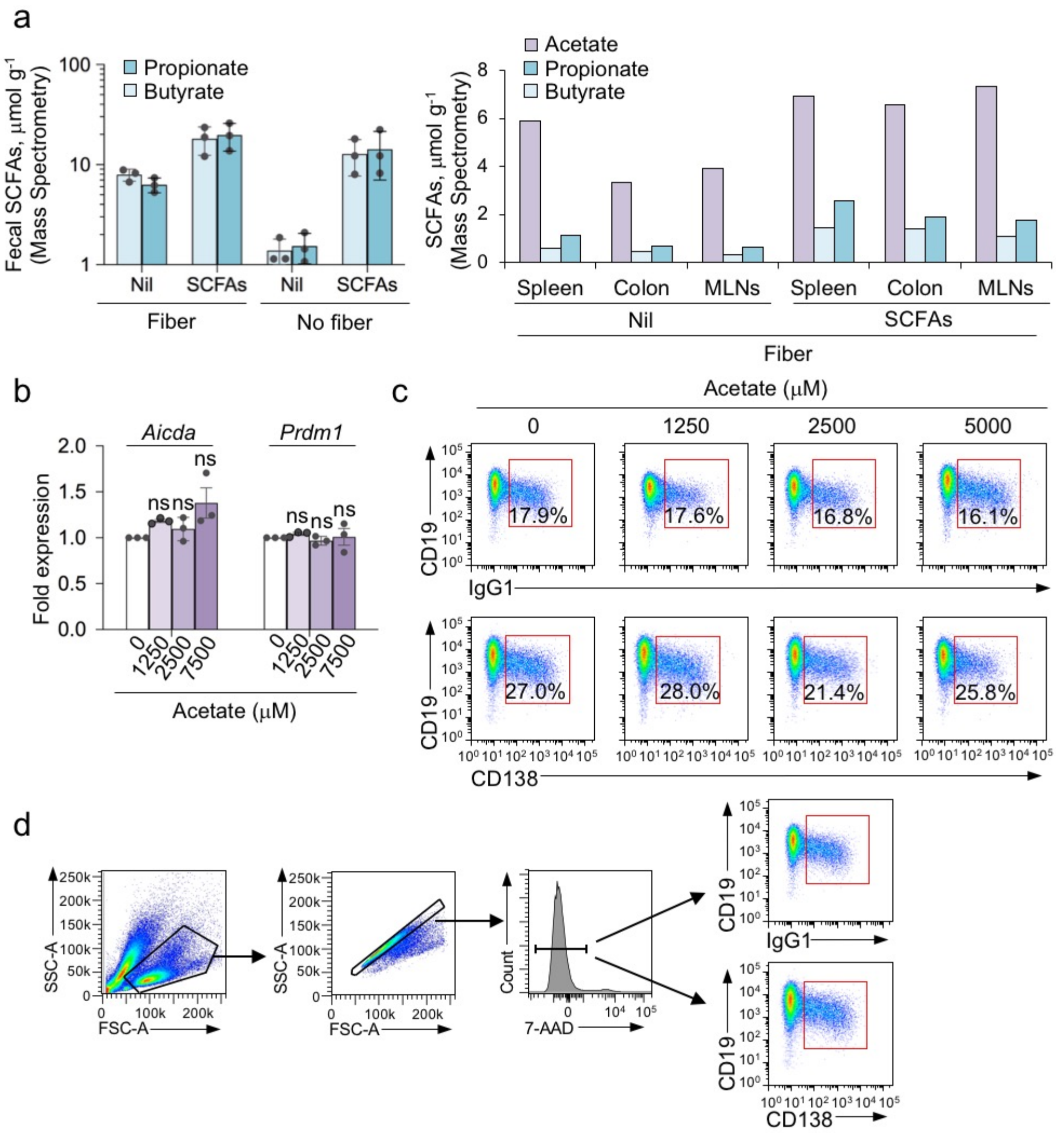

Supplementary Figure 1. Administration of dietary fiber and/or exogenous SCFAs alter gut butyrate and propionate concentrations. Acetate does not alter *Aicda* and *Prdm1* expression, CSR or plasma cell differentiation. **a,** C57BL/6 mice, after weaning, were fed a fiber or no-fiber diet, and given SCFA-water (SCFAs) or plain water (Nil) starting at the age of 5 weeks. Concentrations of butyrate and propionate in the feces, as well as acetate, butyrate and propionate in the spleen, colon and mesenteric lymph nodes were measured by mass spectrometry 21 days later. Fecal and spleen SCFA data are means and SE from 3 mice in each group. Colon SCFA data are means and SE from two mice in each group. MLN SCFA data are from pooled samples of five mice of each group. **b**,**c,** Mouse B cells were stimulated with LPS plus IL-4, in the presence of increased concentration of acetate (0, 1250, 2500 or 5000 μM). The expression of *Aicda* and *Prdm1* transcripts was analyzed 72 h post stimulation by qRT-PCR and normalized to *Gapdh* transcripts. Data are from three independent experiments (mean and SE) (**b**). The proportions of $IgG1^{+}$ B cells and $CD138^{+}$ plasmablasts/plasma cells were analyzed 96 h post stimulation by flow cytometry. Data are one representative of three independent experiments yielding comparable results (**c**). **d,** Gating strategy to analyze class-switched B cells and plasma cells in *in vitro* stimulated cells presented on (**c**). The same strategy was used for similar analysis presented on Fig. 7b,d-f, 5a-d,f and Supplementary Fig. 6a,b, 9a, 10c,d and 11b. The source data are provided in a Source Data file.

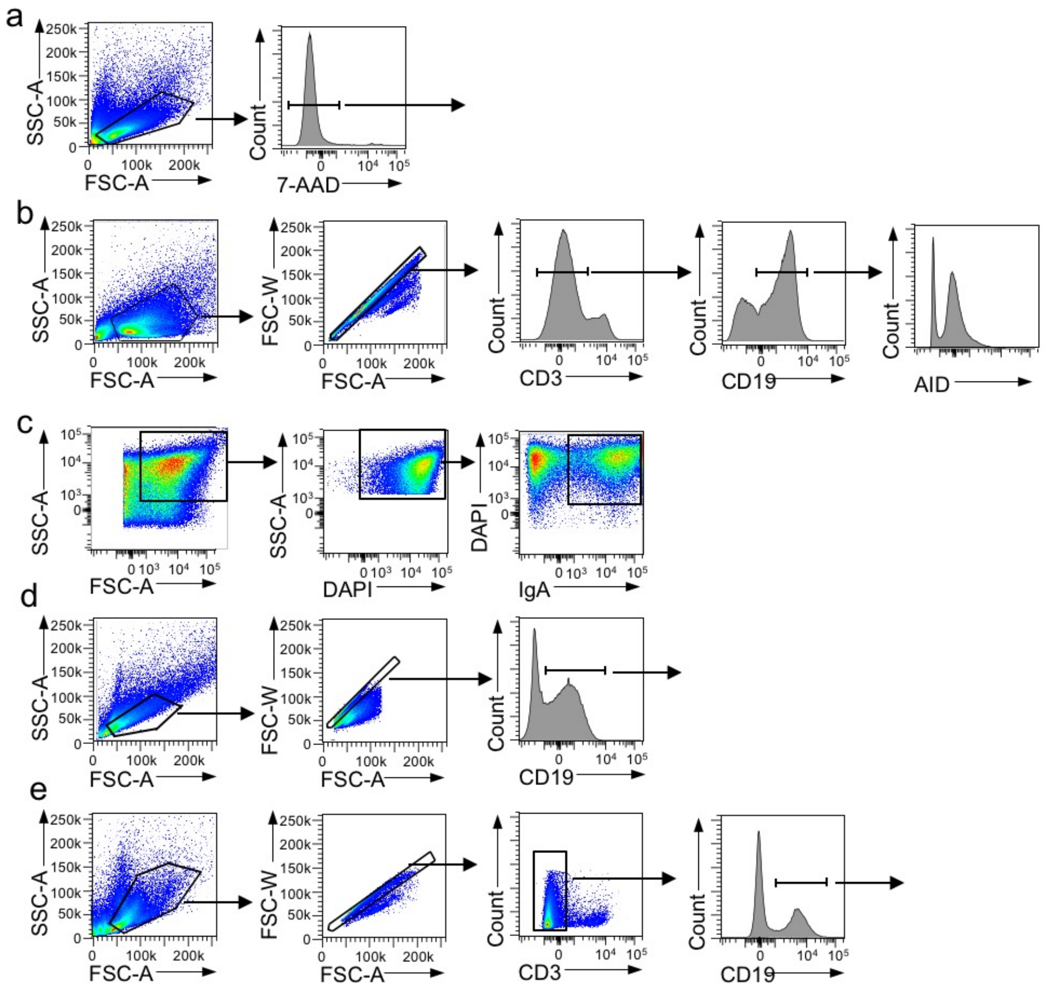

Supplementary Figure 2. Gating strategy for flow cytometry analysis. a, Gating strategy to analyze *ex vivo* class switched B cells, germinal center B cells and plasma cells presented in Fig. 1e. The same strategy was used for similar analysis presented in Fig. 4h. **b**. Gating strategy to analyze intracellular AID expression presented in Fig. 2b. The same strategy was used for similar analysis presented on Fig. 4b, 10d and Supplementary Fig. 13c. **c**, Gating strategy to analyze fecal bacteria-bound antibodies presented in Fig. 2d,e. **d**, Gating strategy to analyze intracellular AID and Blimp1 expression presented in Fig. 3h. **e**, Gating strategy to analyze *ex vivo* class-switched B cells and plasma cells presented in Fig. 4c.

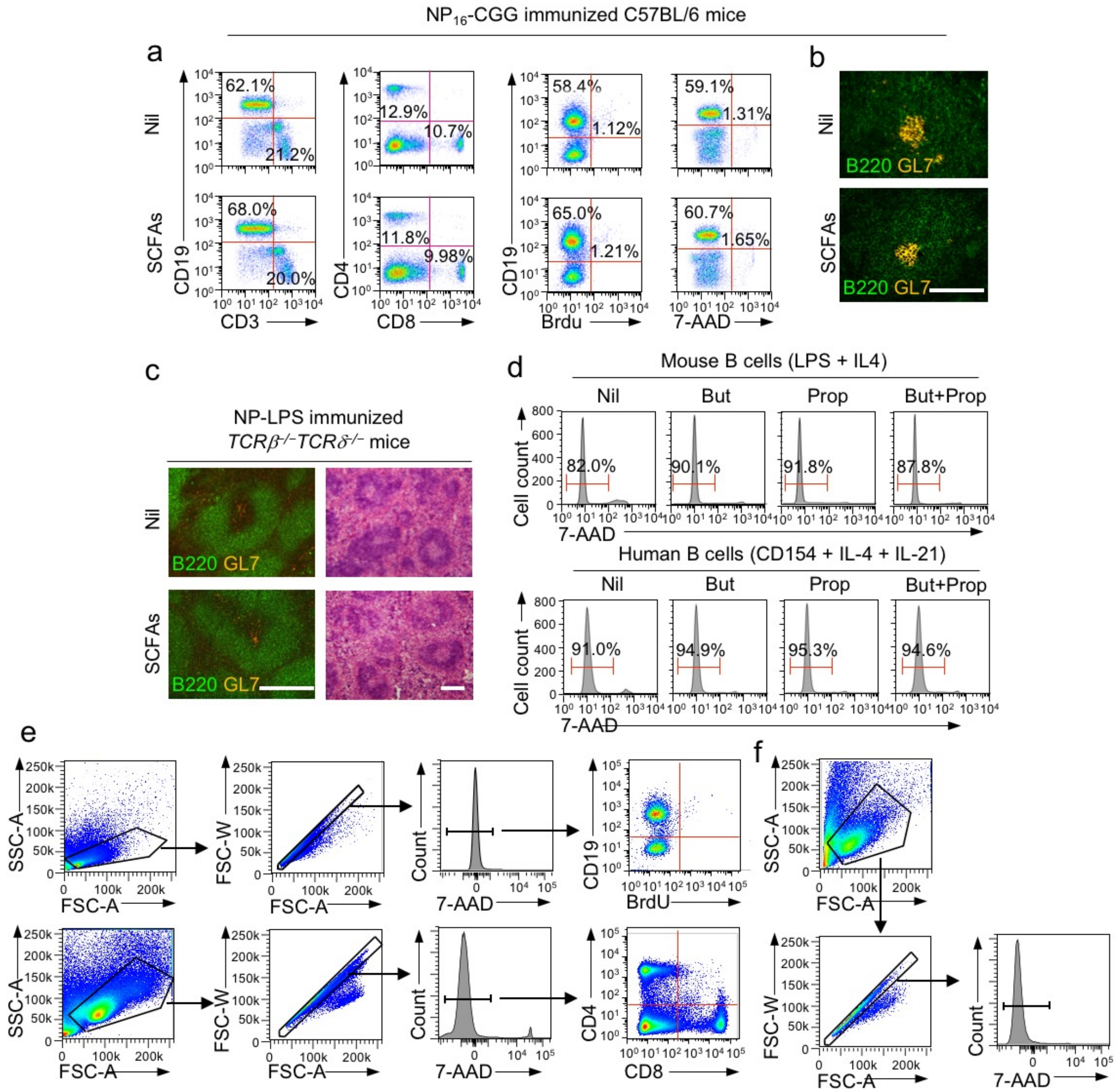

Supplementary Figure 3. SCFAs do not alter B and T cell numbers, B cell proliferation and viability or GC formation. **a**,**b**, C57BL/6 mice fed fiber diet were given SCFA-water (SCFAs) or plain water (Nil) starting at the age of 5 weeks and injected with NP$_{16}$-CGG (in alum) at the age of 8 weeks. The mice were sacrificed 10 days post-injection. Proportions of CD19^{+} B cells, CD3^{+} T cells, CD4^{+} and CD8^{+} T cells, proliferating B cells (BrdU-stained CD19^{+} B cells) and viable (7-AAD^{-}) CD19^{+} B cells in the spleen were analyzed by flow cytometry (**a**). GCs were visualized by fluorescent microscopy (B220, green; GL-7, orange) (**b**). **c**, *Tcrβ$^{-/-}$Tcrδ$^{-/-}$* mice fed fiber diet were given SCFA-water or plain water starting at the age of 5 weeks and injected NP-LPS (in PBS) at the age of 8 weeks. GCs were visualized by fluorescent microscopy (B220, green; GL-7, orange) and H&E staining 10 days post-injection. **d**, Mouse and human B cells were stimulated with LPS plus IL-4 or CD154 plus IL-4 and IL-21, respectively, for 96 h, in the presence of nil, butyrate (500 μM), propionate (2000 μM) or butyrate (500 μM) plus propionate (2000 μM), and analyzed for viable (7-AAD^{-}) CD19^{+} B cells by flow cytometry. Data are one representative of three independent experiments yielding comparable results. **a,** Gating strategy to analyze *ex vivo* B cell proliferation and T cells presented on Supplementary Fig. 3a. **e,** Gating strategy for (**a**). **f,** Gating strategy for (**d**). Scale bar = 100 μm.

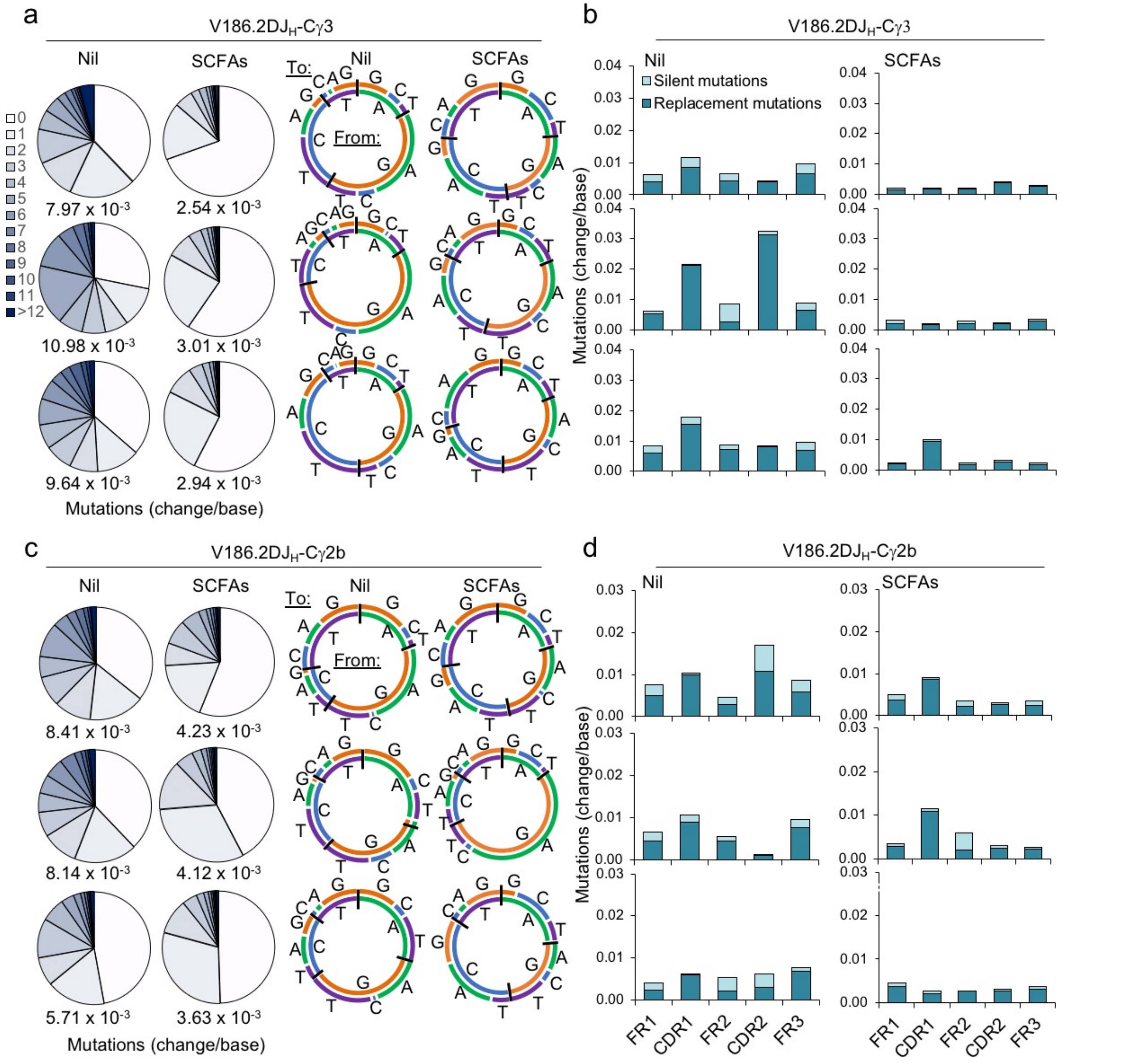

Supplementary Figure 4. SCFAs dampen SHM in the T-independent antibody response to NP-LPS in $Tcr\beta^{-/-}Tcr\delta^{-/-}$ mice. $Tcr\beta^{-/-}Tcr\delta^{-/-}$ mice fed fiber diet were given SCFA-water (SCFAs) or plain water (Nil) starting at the age of 5 weeks and injected with NP-LPS (in PBS) at the age of 8 weeks. The mice were boost-injected 21 days later with NP-LPS (in PBS). Somatic point-mutations were analyzed in the $V_{186.2}$ region of $V_{186.2}DJ_H$-Cγ3 (**a,b**) and $V_{186.2}DJ_H$-Cγ2b (**c,d**) transcripts amplified from spleen B cells 7 days after the boost injection. Pie charts depict the proportions of sequences carrying different numbers of point-mutations over the 294 bp $V_{186.2}$ segment. Listed below the pie charts is the overall mutation frequency (change/base). Donut charts depict the spectrum of point-mutations (**a,c**). Histograms depict frequencies of silent and replacement mutations in FR and CDR regions (**b,d**). Data are from three pairs of fiber fed mice given SCFA-water and plain-water. The source data are provided in a Source Data file.

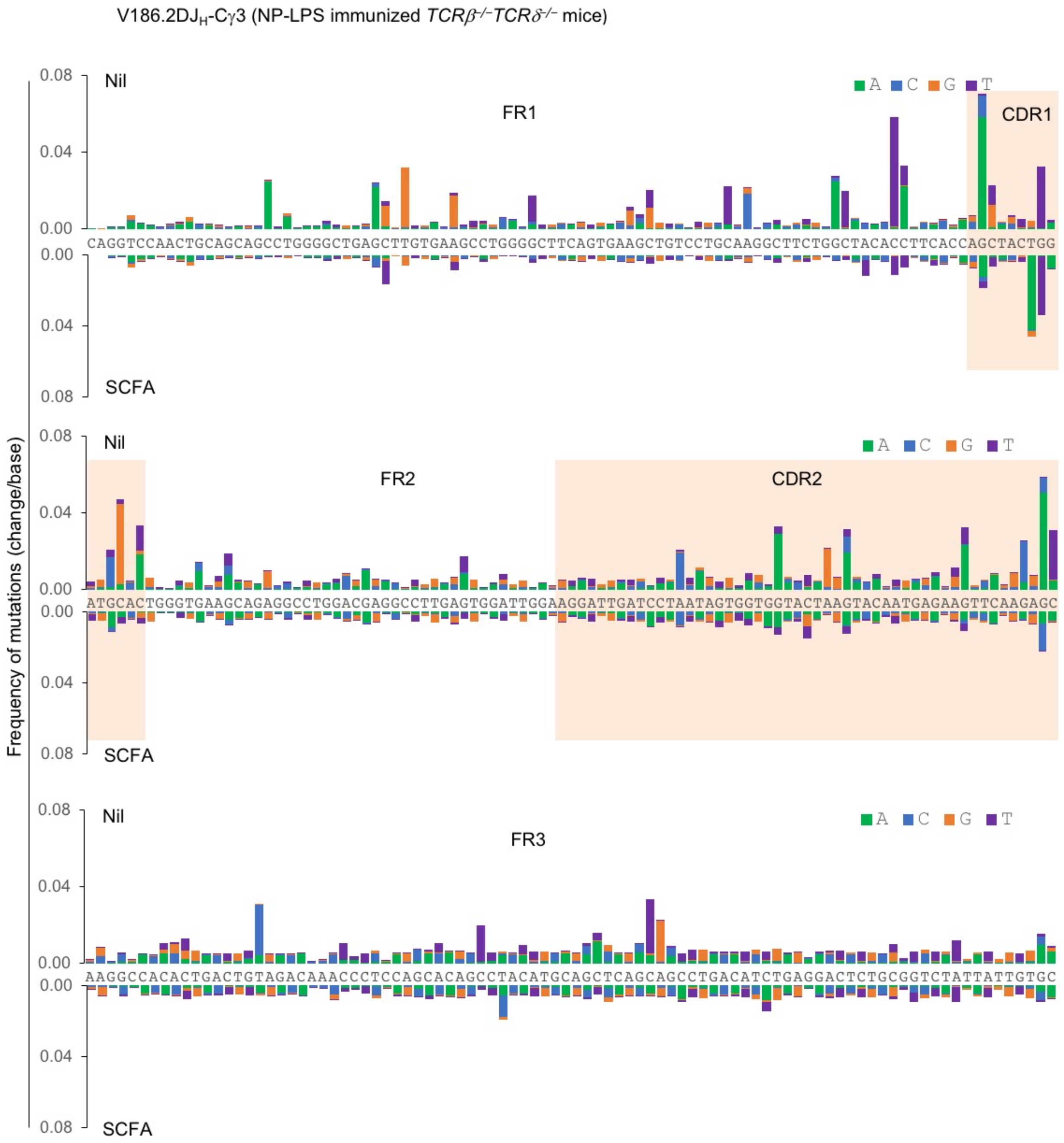

Supplementary Figure 5. Spectrum and distribution of point-mutations in the $V_{186.2}$ region of $V_{186.2}DJ_H$-Cγ3 transcripts in the NP-LPS immunized *Tcrβ*$^{-/-}$*Tcrδ*$^{-/-}$ mice treated with nil or SCFAs. *Tcrβ*$^{-/-}$*Tcrδ*$^{-/-}$ mice fed fiber diet were given SCFA-water (SCFAs) or plain water (Nil) starting at the age of 5 weeks and injected with NP-LPS (in PBS) at the age of 8 weeks. The mice were boost-injected 21 days later with NP-LPS (in PBS). The $V_{186.2}$ region of $V_{186.2}DJ_H$-Cγ3 transcripts were amplified from spleen B cells 7 days after the NP-LPS boost injection and analyzed for spectrum and distribution of point-mutations. Data are pooled from three pairs of fiber-fed mice given SCFA-water or plain water (as in **Supplementary Figure 5**). . The source data are provided in a Source Data file.

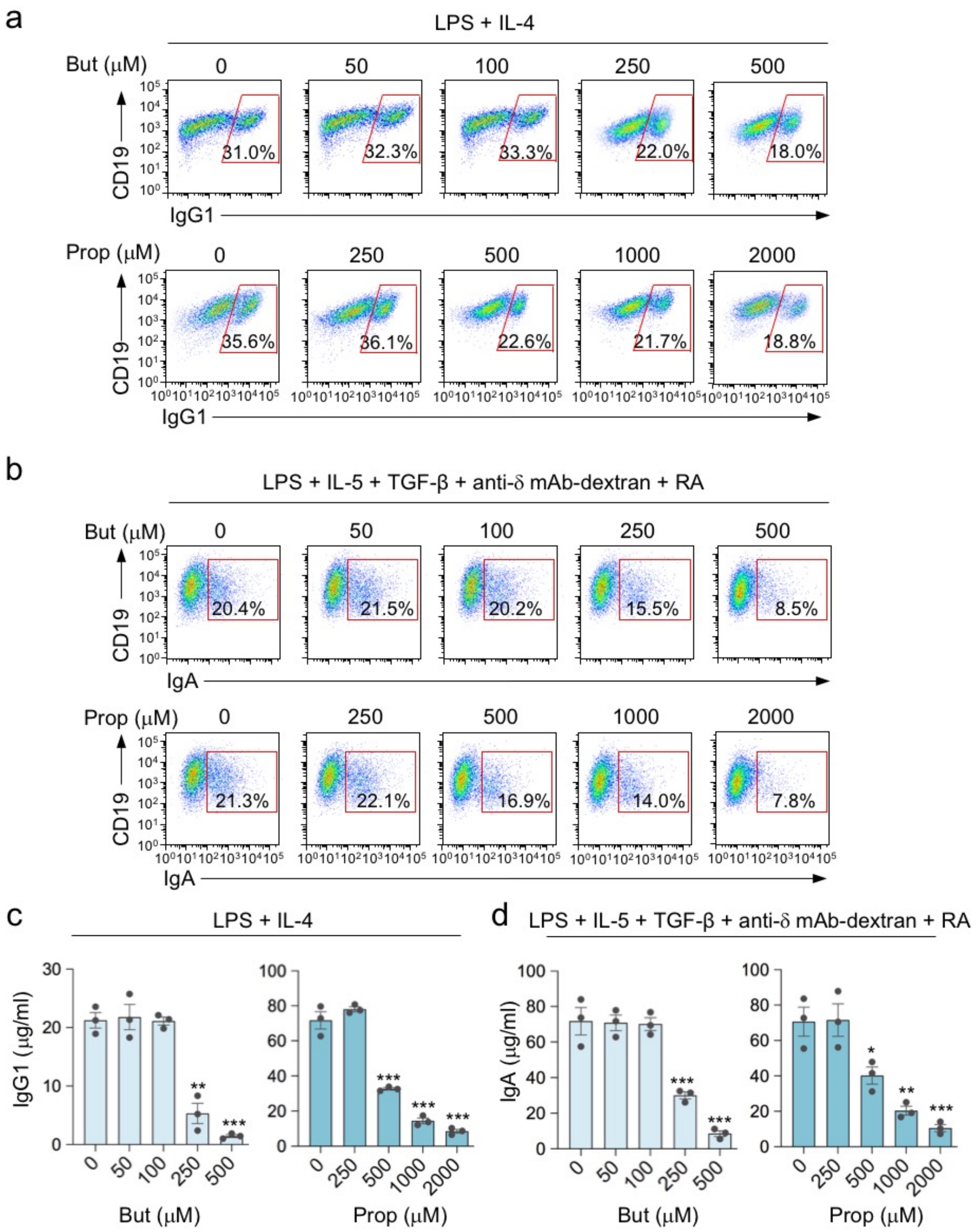

Supplementary Figure 6. Butyrate and propionate inhibit CSR in a dose-dependent fashion. Mouse B cells were stimulated for 96 h with LPS plus IL-4, LPS plus IL-5, TGF-β, anti-δ mAb-dextran and RA, in the presence of nil, butyrate (50, 100, 250 or 500 μM) or propionate (250, 500, 1000 or 2000 μM). **a,b**, The proportions of $IgG1^+$ and IgA^+ B cells were analyzed by flow cytometry. Data are one representative of three independent experiments yielding comparable results. **c,d**, IgG1 and IgA titers in culture fluids as analyzed by specific ELISAs. Data are from three independent experiments (mean and SE). $*p < 0.05$, $**p < 0.01$, $***p < 0.001$ (unpaired *t*-test). . The source data are provided in a Source Data file.

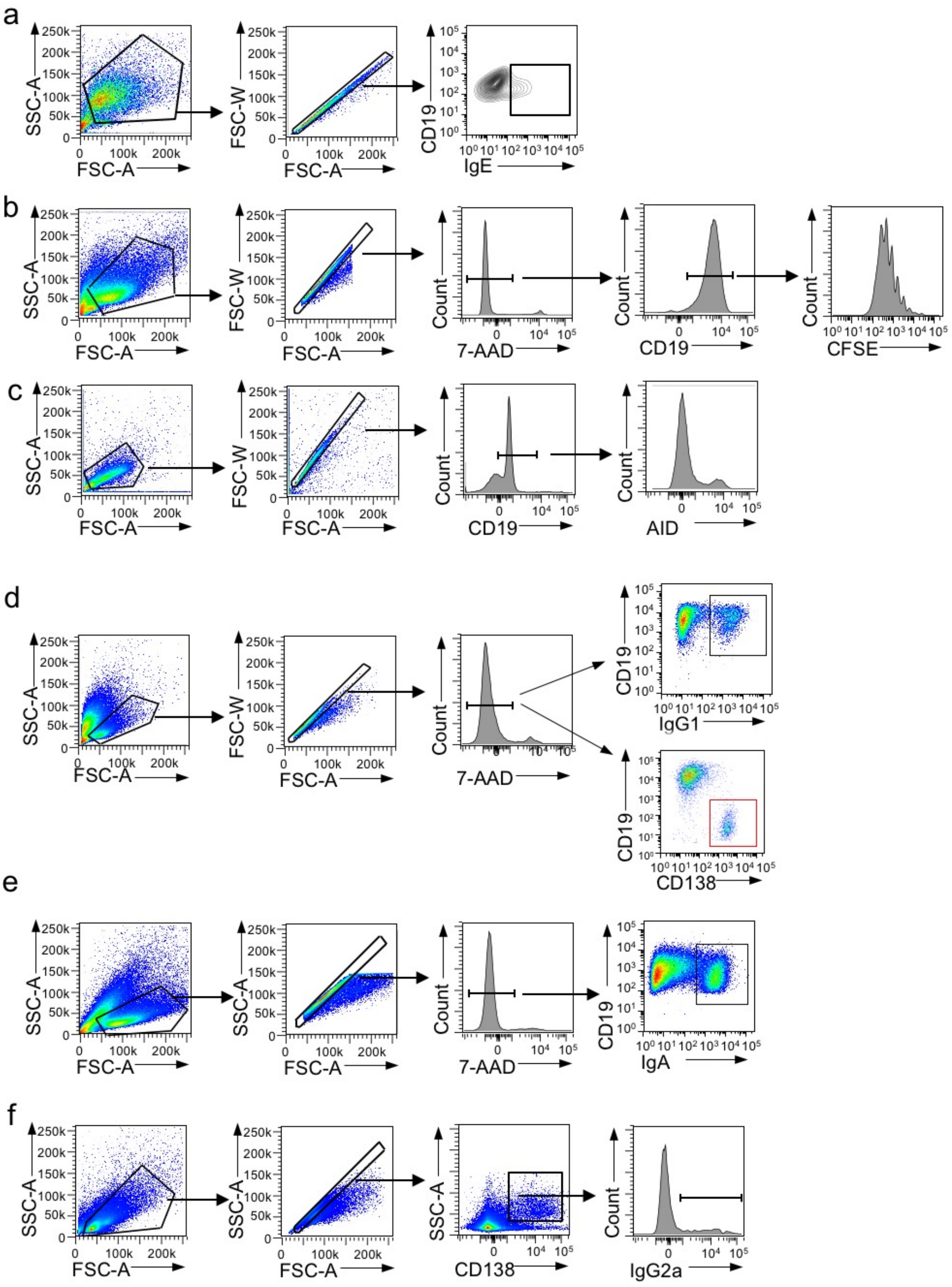

Supplementary Figure 7. Gating strategy for flow cytometry analysis. a, Gating strategy to analyze intracellular intracellular CD19$^+$ IgE$^+$ B cells presented on Fig. 5e. **b,** Gating strategy to analyze CFSE signal in B cells presented on Fig. 5g. **c,** Gating strategy to analyze intracellular AID expression in *in vitro* stimulated B cells presented on Fig. 5k. **d,** Gating strategy to analyze class-switched human B cells and plasma cells presented on Fig. 6a,b. **e,** Gating strategy to analyze IgA+ CH12 B cells presented on Fig. 9f. **f,** Gating strategy to analyze class-switched plasmablasts presented on Supplementary Fig. 13e.

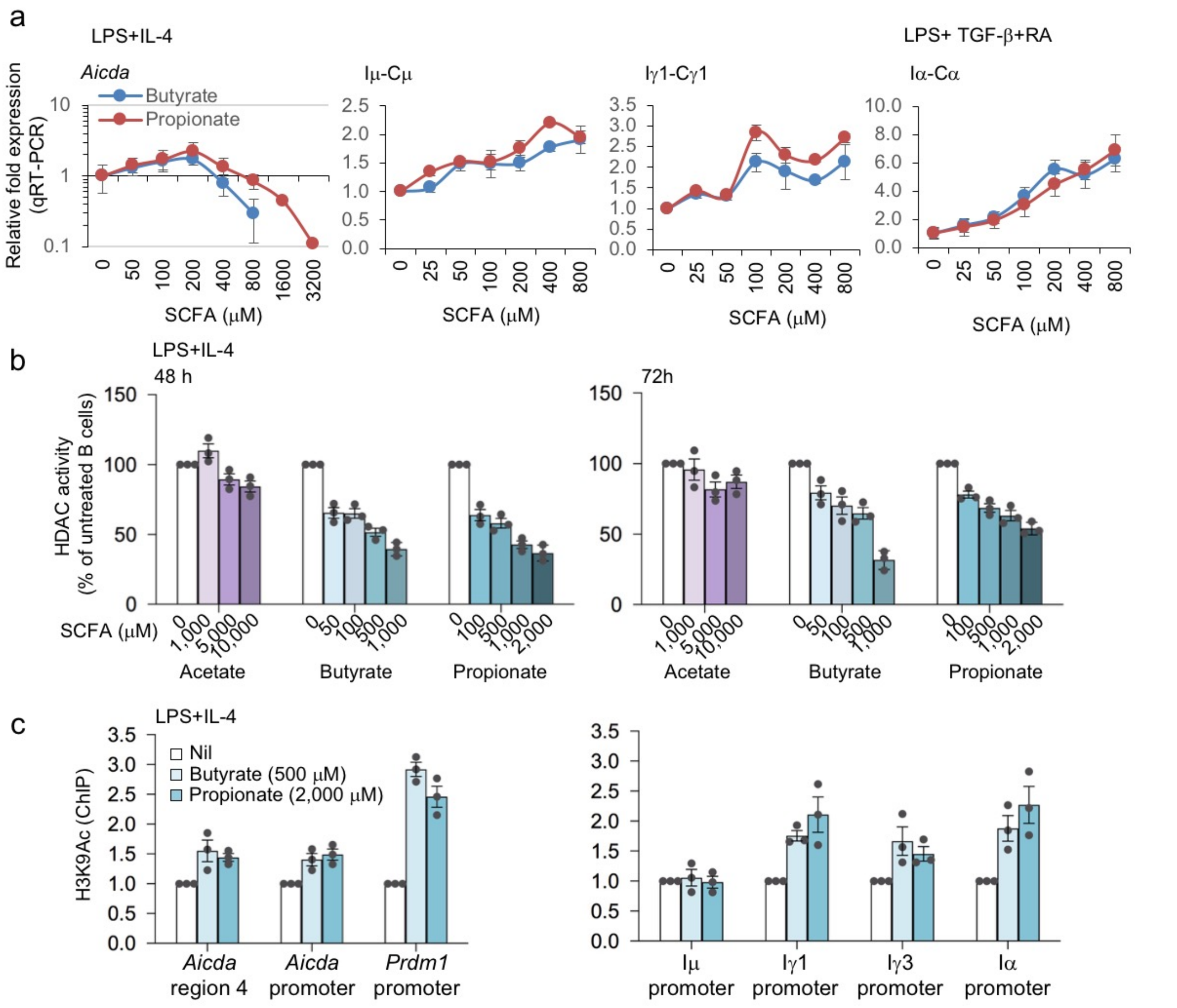

Supplementary Figure 8. Dose-dependent effect of SCFAs in the modulation of *Aicda* and germline I_H-C_H expression and HDAC activity in B cells. a, B cells were stimulated with LPS plus IL-4, or LPS plus TGF-β and RA in the presence of increased dose of butyrate or propionate for 60 h. Expression of *Aicda* and germline Iμ-Cμ, Iγ1-Cγ1 and Iα-Cα transcripts, as analyzed by qRT-PCR and normalized to *β-Actin* transcripts. Data are from 3 independent experiments (mean and SE). **b,** HDAC activity in B cells stimulated with LPS plus IL-4 in the presence of increased dose of acetate, butyrate or propionate for 48 or 72 h. **c,** Acetylated histone H3 (H3K9ac) in the *Aicda* regulatory region 4, *Aicda* promoter, *Prdm1* promoter, Iμ, Iγ1, Iγ3 and Iα promoters in B cells stimulated for 60 h with LPS plus IL-4 (*Aicda* regulatory region 4, *Aicda* promoter, *Prdm1* promoter, Im promoter and Iγ1 promoter), LPS only (Iγ3 promoter) or LPS, plus TGF-β and RA (Iα promoter), as analyzed by ChIP and qPCR. The source data are provided in a Source Data file.

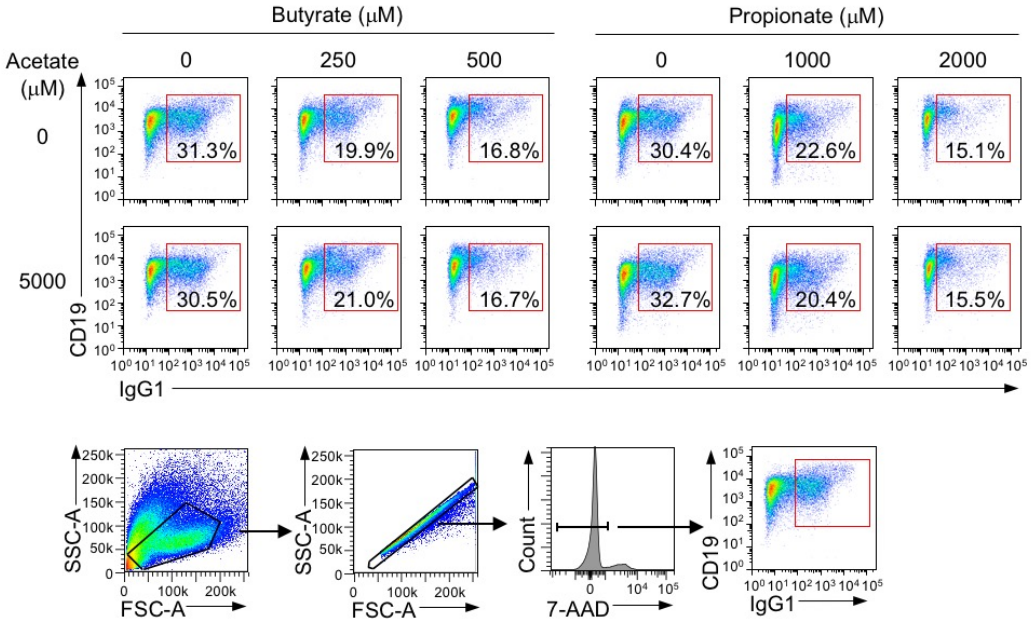

Supplementary Figure 9. Acetate does not alter butyrate- and propionate-mediated inhibition of CSR and plasma cell differentiation. Mouse B cells were stimulated with LPS plus IL-4, and cultured for 96 h with increased concentration of butyrate (0, 250 or 500 μM) or propionate (0, 1000 or 2000 μM) without or with acetate (5000 μM). The proportions of IgG1^{+} B cells were analyzed by flow cytometry. Bottom panels show the gating strategy. Data are one representative of three independent experiments yielding comparable results.

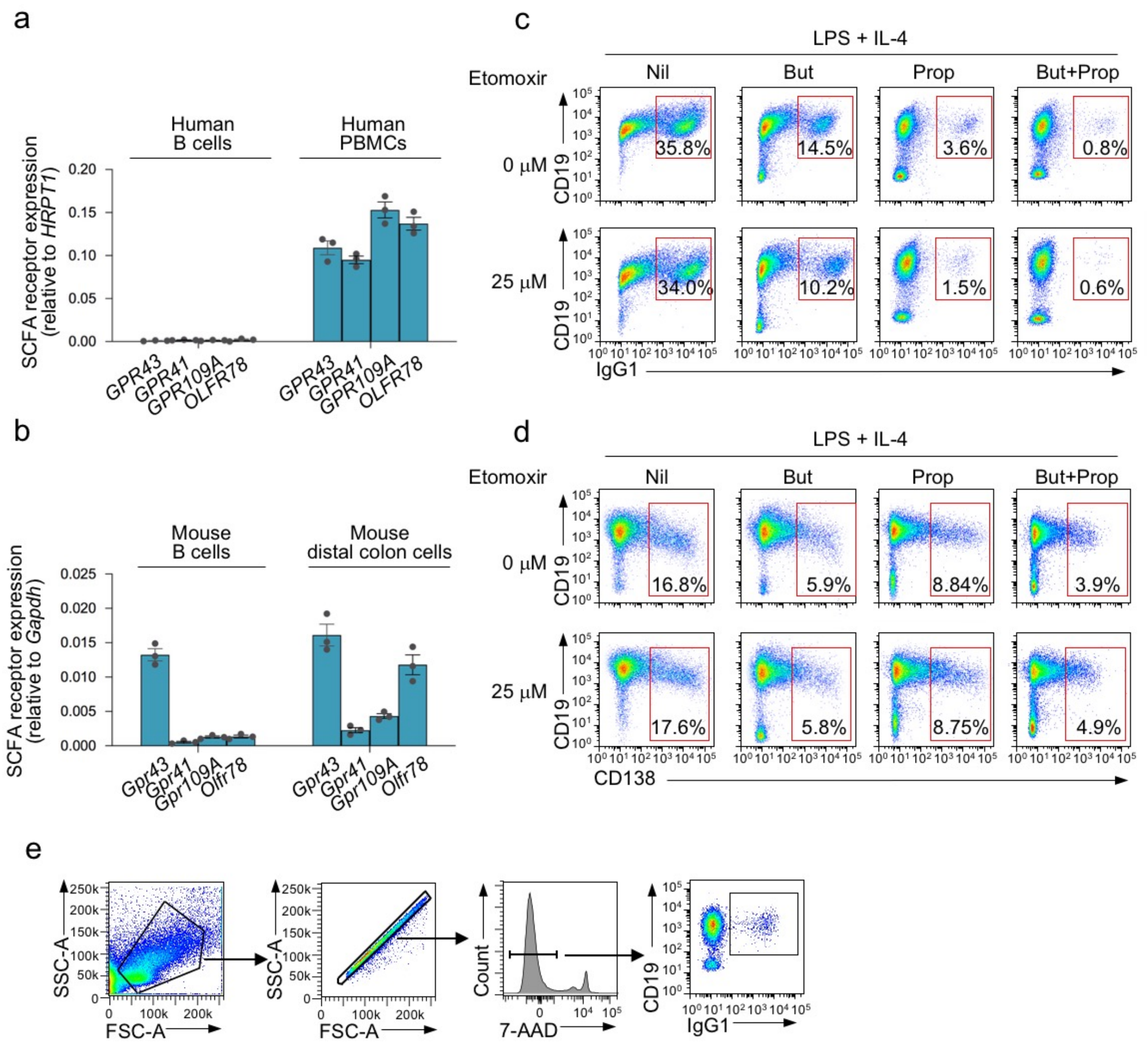

Supplementary Figure 10. Lack of expression of SCFA receptors in human and mouse B cells. Blocking the function of SCFAs as an energy substance by fatty acid β-oxidation inhibitor etomoxir does not affect SCFA-mediated inhibition of CSR and plasma cell differentiation. a, *FFAR2, FFAR3, GPR109A* and *OLFR78* transcripts in human B cells stimulated for 60 h with CD154, IL-21, and IL-4, and human PBMCs were measured by qRT-PCR and normalized to *HRPT* expression. **b**, *Ffar2, Ffar3, Gpr109A* and *Olfr78* transcripts in mouse B cells stimulated with LPS plus IL-4 for 60 h and mouse distal colon cells were measured by qRT-PCR and normalized to *β-Actin* expression. Data are mean and SE from three independent experiments. **c,d,** Mouse B cells were cultured with LPS plus IL-4 in the presence of nil, butyrate (500 μM), propionate (2000 μM) or butyrate (500 μM) plus propionate (2000 μM), without or with etomoxir (25 μM). After 96 h, IgG1$^+$ B cells (**c**) and CD138$^+$ plasmablasts/plasma cells (**d**) were analyzed by flow cytometry. Data are one representative of three independent experiments yielding comparable results. **e**, Gating strategy for (**c**) and (**d**). The source data are provided in a Source Data file.

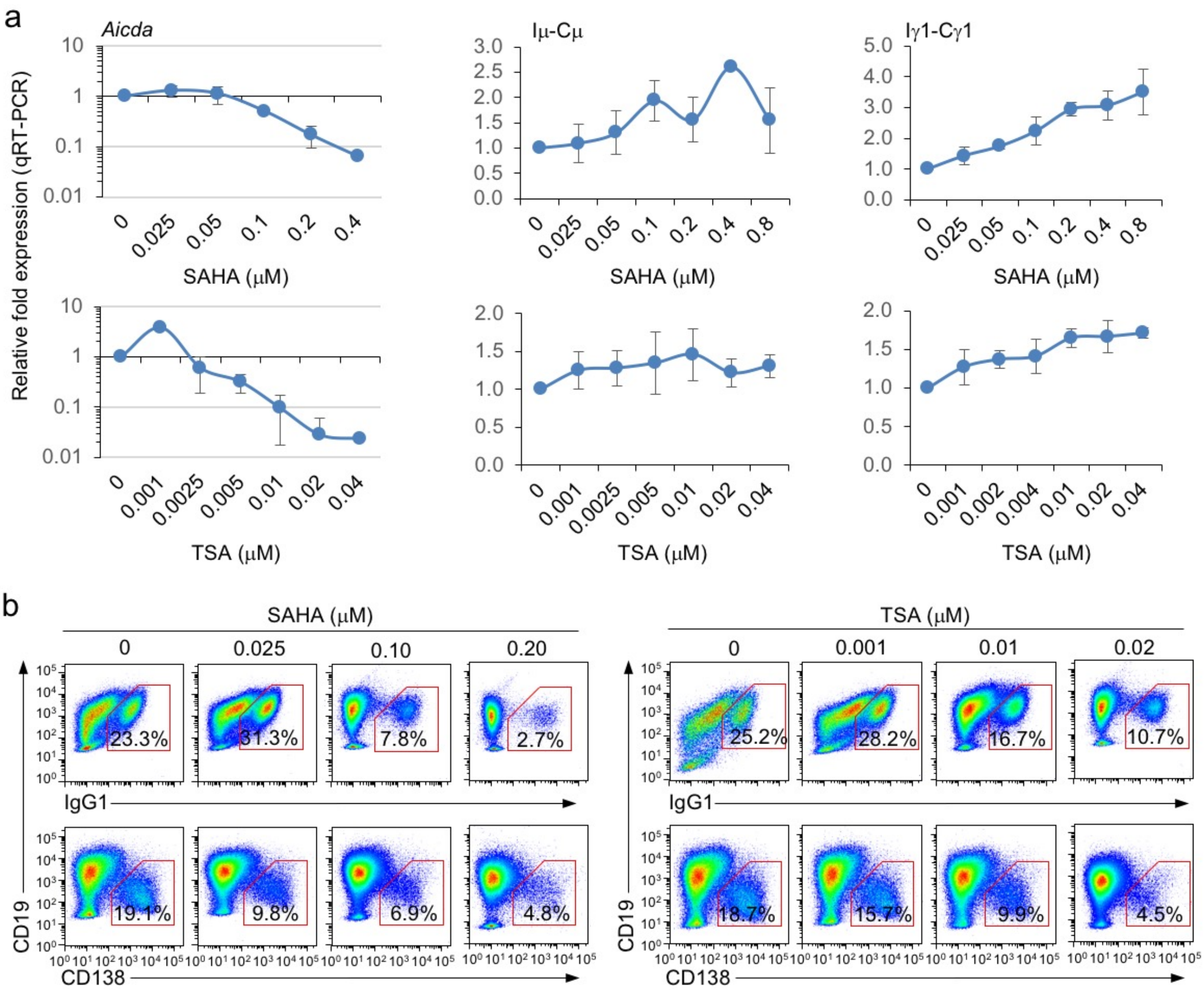

Supplementary Figure 11. Dose-dependent effect of HDAC inhibitors SAHA and TSA in the modulation of *Aicda* and germline I_H-C_H expression, CSR and plasma cell differentiation. B cells were stimulated with LPS plus IL-4 in the presence of increased dose of SAHA or TSA. **a**, Expression of *Aicda* and germline Iμ-Cμ and Iγ1-Cγ1 transcripts, as analyzed 60 h post stimulation by qRT-PCR and normalized to *β-Actin* transcripts. Data are from 3 independent experiments (mean and SE). **b,** The proportions of $CD19^+$ $IgG1^+$ B cells and CD19lowCD138+ plasma cells were analyzed 96 h post stimulation by flow cytometry. Data are representative of 3 independent experiments yielding comparable results. The source data are provided in a Source Data file.

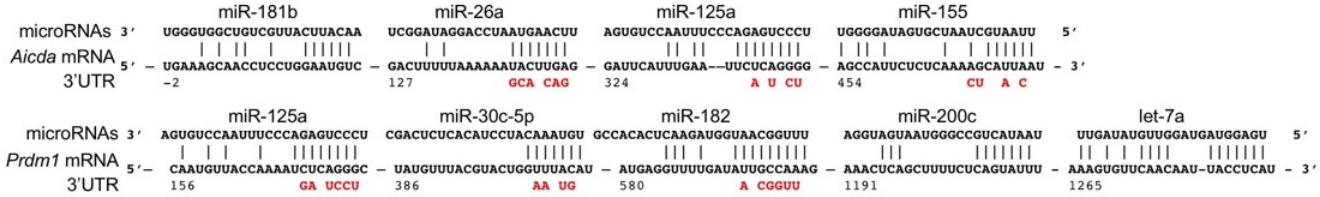

Supplementary Figure 12. Alignment of the 3′ UTRs of *Aicda* and *Prdm1* mRNAs and the targeting miRNAs. The nucleotide changes in the mutant *Aicda* and *Prdm1* 3′ UTRs used in luciferase reporter constructs depicted in red.

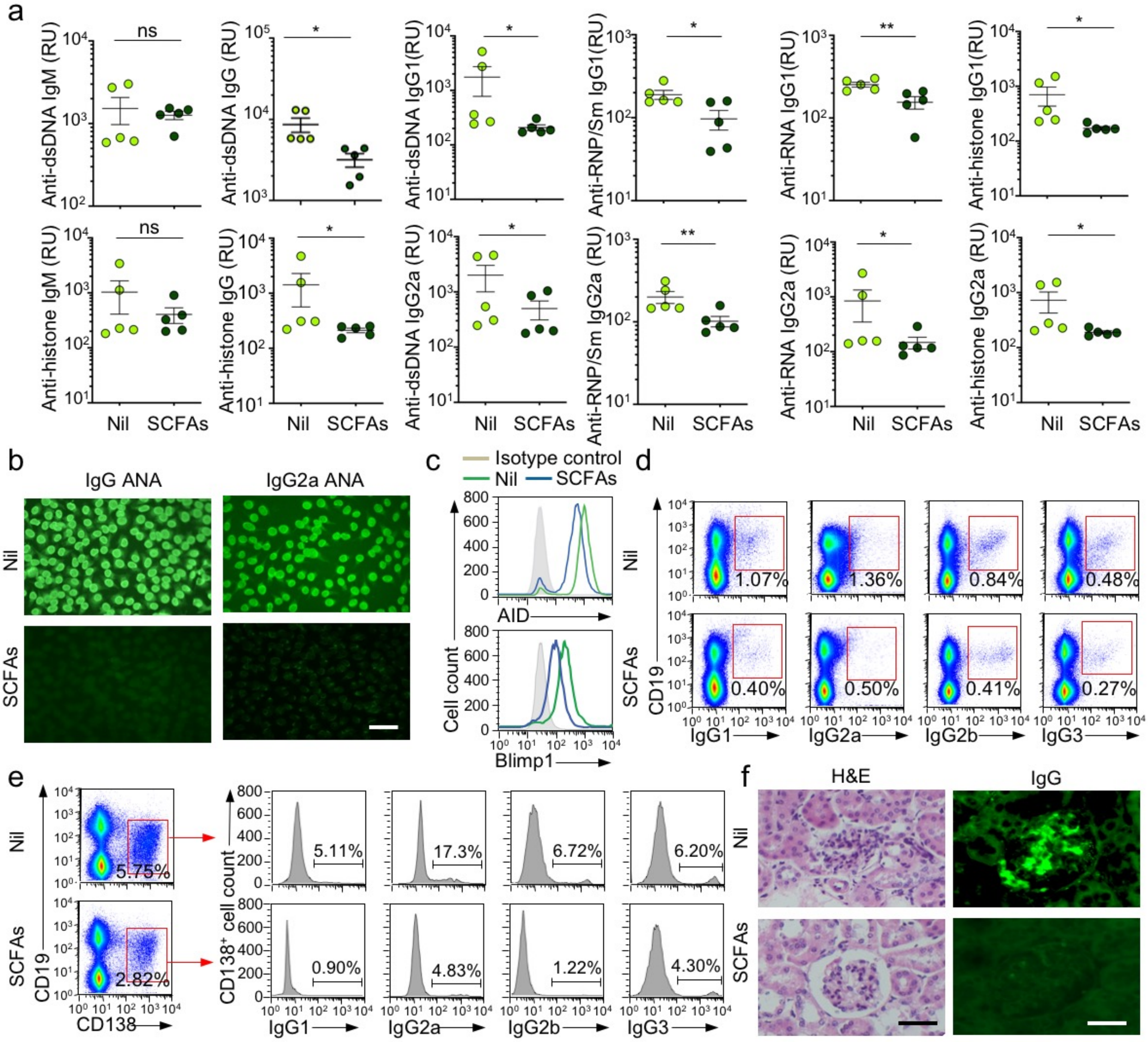

Supplementary Figure 13. SCFAs reduce autoantibody response and autoimmunity in lupus-prone NZB/W F1 mice. Female NZB/W F1 mice fed fiber diet were given SCFA-water (SCFAs) or plain water (Nil) starting at the age of 14 weeks and sacrificed at 26 weeks of age. **a,** Titers of circulating anti-dsDNA IgM, and anti-dsDNA, anti-RNP/Sm, anti-histone and anti-RNA IgG1 and IgG2a (relative units, RU), as analyzed by specific ELISAs. Each symbol represents an individual mouse (n = 5 per group). *$p < 0.05$, **$p < 0.01$, ns: not significant (unpaired t-test). **b,** ANA visualized by indirect immunofluorescence on HEp-2 cells that were incubated with sera (1:400 dilution) from the NZB/WF1 mice using FITC-labeled rat mAbs to mouse IgG and IgG2a. Data are from one of 3 independent experiments yielding comparable results. **c,** AID and Blimp-1 expression as analyzed by intracellular staining followed by flow cytometry. **d**, Spleen surface IgG1, IgG2a, IgG2b and IgG3 B cells, and **e**, intracellular CD19$^{low/neg}$CD138^{+}, CD138^{+} IgG1^{+}, CD138^{+} IgG2a^{+}, CD138^{+} IgG2b^{+}, and CD138^{+} IgG3^{+} plasmablasts/plasma cells as analyzed by flow cytometry. Data are one representative of 3 independent experiments yielding comparable results. **f,** Kidney sections stained with H&E (right panels), and kidney sections stained with FITC-labeled rat mAbs to mouse IgG (right panels). Data are one representative of 3 independent experiments. Scale bar = 20 μm (**b**) or 100 μm (**f**) . The source data are provided in a Source Data file.

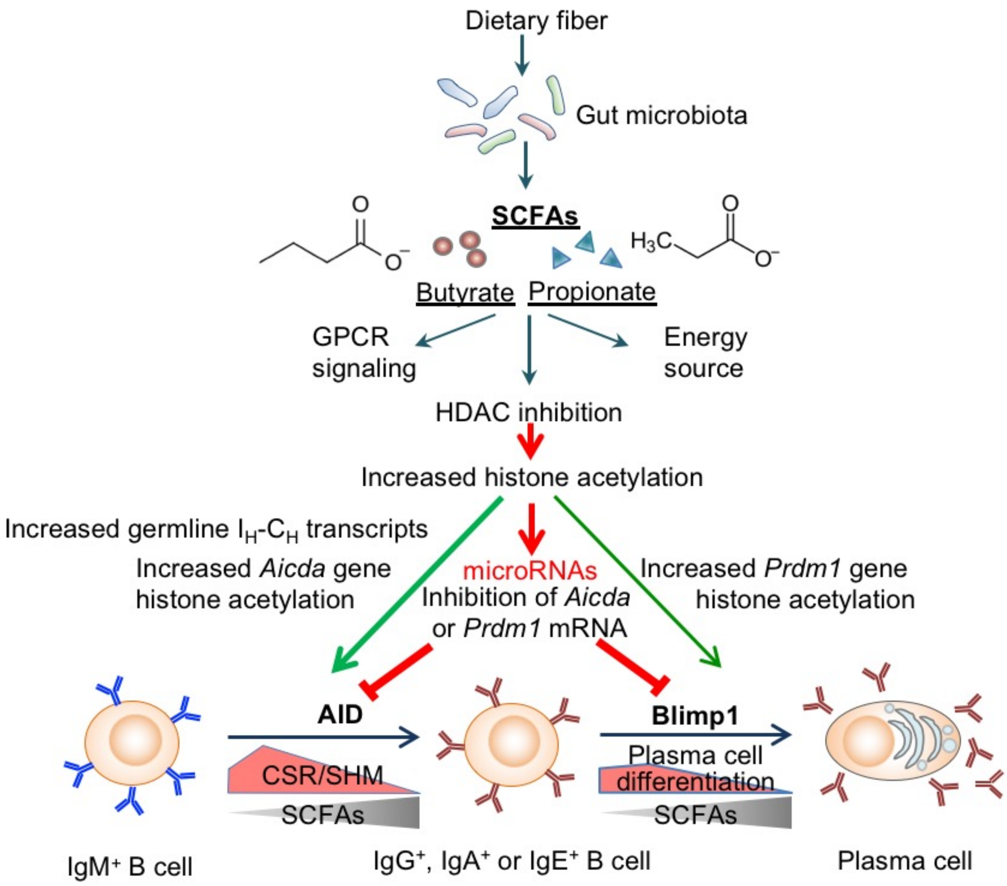

Supplementary Figure 14. Dose-dependent modulation of *Aicda* and *Prdm1* expression, CSR, SHM and plasma cell differentiation by SCFAs. SCFAs butyrate and propionate modulate B cell functions through HDAC inhibition activity, which leads to increased histone acetylation of many genes, including the host genes of select miRNAs targeting *Aicda* or *Prdm1*. While *Aicda*- and *Prdm1*-targeting miRNAs induced by SCFAs inhibits *Aicda* and *Prdm1* in a dose-dependent fashion over a wide physiological SCFAs range, the *Aicda*- and *Prdm1*-targeting miRNAs-mediated inhibition of *Aicda* expression by low-dose SCFAs is not robust enough to override the enhancement of CSR/SHM resulting from increased SCFAs-mediated histone acetylation and germline I_H-C_H transcription. As for *Prdm1*, low-dose SCFAs merely are not sufficient to significantly dampen this gene expression, and, therefore, Blimp1 expression and plasma cell differentiation.

Supplementary Table 1. Antibodies used for this study.

Antibody to	Type	Company	Cat. No. (mAb clone)	Assays
Mouse B220, FITC-conjugated	Rat mAb	BioLegend	103206	FCM
Mouse CD19, Pacific Blue-conjugated	Rat mAb	BioLegend	115523 (6D5)	FCM
Mouse IgM, PE-conjugated	Rat mAb	Biolegend	406507	FCM
Mouse IgG1, FITC-conjugated	Rat mAb	BD Biosciences	553443 (A85-1)	FCM, ANA, IC
Mouse IgG1, APC-conjugated	Rat mAb	BioLegend	406609	FCM, IF
Mouse IgG2a, FITC-conjugated	Rat mAb	BD Biosciences	553390 (R19-15)	FCM, ANA, IC
Mouse IgD, FITC-conjugated	Rat mAb	BioLegend	405713	FCM, IF
Mouse IgA, FITC-conjugated	Rat mAb	BD Biosciences	559354 (C10-3)	FCM
Mouse IgM, PE-conjugated	Goat mAb	BioLegend	Dec-12	FCM, IF
Mouse CD138, PE-Cy7-conjugated	Rat mAb	BioLegend	142514 (281-2)	FCM
Mouse Blimp1, APC- conjugated	Rat mAb	BioLegend	150007 (5 E7)	FCM
Mouse AID, FITC- conjugated	Rabbit pAb	Bioss	bs-7855R-FITC	FCM
Mouse CD4, APC-conjugated	Rat mAb	BioLegend	100411	FCM
Mouse CD8, PE-conjugated	Rabbit mAb	BD Biosciences	563234	FCM
Mouse IgA, unconjugated	Rabbit mAb	Thermo Fisher	PA-1-30826	IF
Rabbit-IgG (H+L), Alexa Fluor 488-conjugated	Goat mAb	Cell Signaling	4412	IF
Mouse IgE, FITC-conjugated	Rat mAb	eBioscience	50-995-0 (23G3)	FCM
GL7, PE-conjugated	Rat mAb	BioLegend	144607	FCM, IF
Human CD19, PE-conjugated	Mouse mAb	BioLegend	302208	FCM
Human IgM, FITC- conjugated	Mouse mAb	Biolegend	314506	FCM
Human IgA, FITC- conjugated	Goat pAb	Sigma Aldrich	F5259	FCM
Human IgG, allophycocyanin	Mouse mAb	BD Biosciences	562025	FCM
Human IgM, Biotin- conjugated	Goat pAb	Southern Biotech	2022-08	FCM
Sm/RNP, unconjugated	From Calf thymus	MyBioSource	MBS318132	ELISA
Histone, unconjugated	Histone from calf thymus	Sigma Aldrich	H9250	ELISA
Mouse Ig, unconjugated	Goat pAb	Southern Biotech	1010-08	ELISA, ELISPOT
Mouse IgG, unconjugated	Goat pAb	Southern Biotech	1030-08	ELISA, ELISPOT
Mouse IgE, unconjugated	Goat pAb	Bethyl	A90-1158	ELISA, ELISPOT
Mouse IgM, unconjugated	Goat pAb	Southern Biotech	1020-01	ELISA, ELISPOT
Mouse IgA, unconjugated	Goat pAb	Southern Biotech	1040-01	ELISA, ELISPOT
Mouse IgM, biotin-conjugated	Goat pAb	Southern Biotech	1022-08	ELISA, ELISPOT
Mouse IgG1, biotin-conjugated	Goat pAb	Southern Biotech	1070-08	ELISA, ELISPOT
Mouse IgG2a, biotin-conjugated	Goat pAb	Southern Biotech	1080-08	ELISA, ELISPOT
Mouse IgG2b, biotin-conjugated	Rat mAb	Biolegend	406704 (RMG2b-1)	ELISA
Mouse IgG3, biotin-conjugated	Goat pAb	Southern Biotech	1100-08	ELISA, ELISPOT
Mouse IgE, biotin-conjugated	Goat pAb	Southern Biotech	1110-08	ELISA, ELISPOT
Mouse IgA, biotin-conjugated	Goat pAb	Southern Biotech	1040-08	ELISA, ELISPOT
Ovalbumin, biotin-conjugated		GALAB	152060	ELISA, ELISPOT

EZ link sulfo-NHS-SS-Biotin		Thermo Fisher	21328	ELISA, ELISPOT
Mouse Blimp1	Rabbit mAb	eBioscience	50-166-72 (6D3)	IB
Mouse AID	Mouse mAb	Invitrogen	39-2500 (ZA001)	IB
Mouse acetyl-histone H3 (H3K9ac/K14ac)	Rabbit mAb	Millipore	17-615	IB
Mouse histone 3	Rat mAb	Biolegend	601901	IB
Mouse β-actin	Mouse mAb	Sigma	A5441 (AC-15)	IB
Mouse B220, FITC-conjugated	Rat mAb	BioLegend	103206	FCM
Mouse CD19, Pacific Blue-conjugated	Rat mAb	BioLegend	115523 (6D5)	FCM
Mouse IgM, PE-conjugated	Rat mAb	Biolegend	406507	FCM

Abbreviations: mAb, monoclonal antibody; pAb, polyclonal antibody; ANA, anti-nuclear antibody analysis; ELISA, enzyme-linked immunosorbent assay; ELISPOT, enzyme-linked immunosorbent spot; FCM, flow cytometry; IB, immunoblotting; IC, immunocomplex analysis.

Supplementary Table 2. PCR Primers used for this study.

	Forward primer	Reverse primer
<u>Mouse genes</u>		
Aicda	5'-AGAAAGTCACGCTGGAGACC-3'	5'-CTCCTCTTCACCACGTAGCA-3'
Prdm1	5’-GCTGCTGGGCTGCCTTTGGA-3’	5’-GGAGAGGAGGCCGTTCCCCA-3’
Irf4	5’-AAAGAGCTGACCACGACGAG-3’	5’-AAAGCCCATCTGGAGCCATC-3’
Xbp1	5’-AAGCCCGGATGAGCGAGCTG-3'	5’-ACCCGGCCACCAGCCTTACT-3’
Sdc1	5’-CAGGAAGGAAGTGCTGGGAG-3’	5’-GCTGCCTTCGTCCTTCTTCT-3’
β-Actin	5’-CTAAGGCCAACCGTGAAAG-3’	5’-ACCAGAGGCATACAGGGACA-3’
Gapdh	5′-TTCACCACCATGGAGAAGGC-3′	5′-GGCATGGACTGTGGTCATGA-3′
Cd79b	5′-CCACACTGGTGCTGTCTTCC-3′	5′-GGGCTTCCTTGGAAATTCAG-3′
<u>Human genes</u>		
AICDA	5’-GTCACCTGGTTCACCTCCTG-3’	5’-CTTGCGGTCCTCACAGAAGT-3’
PRDM1	5’-ATCTTGGGGTAAAAGCGGGT-3’	5'-TCCTGCACTACTGGACACAC-3'
β-ACTIN	5’-AGAGCTACGAGCTGCCTGAC-3’	5’-AGCACTGTGTTGGCGTACAG-3’
<u>Germline transcripts</u>		
Mouse		
Iμ-Cμ	5'-ACCTGGGAATGTATGGTTGTGGCTT-3'	5'-GCAGGCAGGGCTAGATATGG-3'
Iγ1-Cγ1	5′-TCGAGAAGCCTGAGGAATGTG-3′	5′-ATGGAGTTAGTTTGGGCAGCA-3′
Iγ3-Cγ3	5'-AACTACTGCTACCACCACCACCAG-3'	5'-AGCCAGGGACCAAGGGATAGAC-3'
Iγ2b-Cγ2b	5'GATGGGGAGGAGTTGGCAGAT-3'	5'-CGGAGGAACCAGTTGTATC-3'
Iα-Cα	5'-GCTTCCTGGAAAGCAGCAAC-3'	5'-TAATCGTGAATCAGGCAG-3'
Iε-Cε	5’-CCCCACTTTTAGCTGAGGGC-3’	5’-ACAGGGCTTCAAGGGGTAGA-3’
Human		
Iγ1-Cγ1	5'-CTTCCAAGCCAACAGGGCAG-3'	5'-ACCTGTGAGGTGGCTGCGTACTT-3'
Iα-Cα	5'-GCCATCAAGGCAGGGCCTGGG-3'	5'-TGCGACGACCACGTTCCCATCTTGGG-3'
Iε-Cε	5'-GACGGGCCACACCATCC-3'	5'-CGGAGGTGGCATTGGAGG-3'
<u>Mouse post-recombination transcripts</u>		
Iμ-Cγ1	5'-ACCTGGGAATGTATGGTTGTGGCTT-3’	5′-ATGGAGTTAGTTTGGGCAGCA-3′
Iμ-Cγ2a	5'-ACCTGGGAATGTATGGTTGTGGCTT-3'	5'-GCTGGGCCAGGTGCTCGAGGTT-3'
Iμ-Cγ2b	5'-ACCTGGGAATGTATGGTTGTGGCTT-3'	5'-CGGAGGAACCAGTTGTATC-3'
Iμ-Cγ3	5'-ACCTGGGAATGTATGGTTGTGGCTT-3'	5'-AGCCAGGGACCAAGGGATAGAC-3'
Iμ-Cα	5'-ACCTGGGAATGTATGGTTGTGGCTT-3'	5'-TAATCGTGAATCAGGCAG-3'
Iμ-Cε	5'-ACCTGGGAATGTATGGTTGTGGCTT-3’	5’-ACAGGGCTTCAAGGGGTAGA-3’
<u>Human mature transcripts</u>		
V_HDJ_H-Cγ1	5'-CAGGTGCAGCTGGTGSARTCTGG-3'	5'-ACCTGTGAGGTGGCTGCGTACTT-3'
V_HDJ_H-Cα	5'-CAGGTGCAGCTGGTGSARTCTGG-3'	5'-TGCGACGACCACGTTCCCATCTTGGG-3'
V_HDJ_H-Cε	5'-CAGGTGCAGCTGGTGSARTCTGG-3'	5'-CGGAGGTGGCATTGGAGG-3'
<u>Mature miRNA and sn/snoRNA forward primers (used with Qiagen miScript Universal Primer as reverse primer)</u>		
miR-155-5p	5’-TTAATGCTAATTGTGATAGG-3’	
miR-181b-5p	5’-AACATTCATTGCTGTCGGTG-3	
miR-26a-5p	5’-TTCAAGTAATCCAGGATAGGCT-3′	
miR-125a-5p	5'-TCCCTGAGACCCTTTAACCTGTGA-3'	
miR-30c-5p	5'-TGTAAACATCCTACACTCTCAGC-3'	
miR-182-5p	5'-TTTGGCAATGGTAGAACTCACACCG-3'	
miR-200c-5p	5'-TAATACTGCCGGGTAATGATGGA-3'	

Mir-let7a-5p	5'-TGAGGTAGTAGGTTGTATAGTT-3'	
miR-19a/b-3p	5’-TGTGCAAATCTATGCAAAACTG-3’	
miR-20a-5p	5’-AAAGTGCTTATAGTGCAGGTAG-3’	
miR-25-3p	5’-CATTGCACTTGTCTCGGTCTG-3’	
miR-24-3p	5’-TGGCTCAGTTCAGCAGGAACAG-3’	
miR-106-5p	5’-CAAAGTGCTAACAGTGCAGGTAG-3’	
miR-27b	5’-TTCACAGTGGCTAAGTTCTGC-3’	
miR-186	5’-CAAAGAATTCTCCTTTTGGGCT-3’	
miR-206-3p	5’-TGGAATGTAAGGAAGTGTGTGG-3’	
miRNA host gene (HG) promoter ChIP		
mir-155-HG	5'-AAGGTCATGAGTTCAAGGCCAGC-3'	5'-TGTGCATGTGTGCATGAGTGCCT-3'
mir-181b-HG	5'-TGTGCATGTGTGCATGAGTGCCT-3'	5'-ACTGCAAGGACGCATGTAGGTCA-3'
mir-26a-HG	5'-CGTCGGAAACACAAACACCC-3'	5'-GTGGCCCCTCCTTACTATGC-3'
mir-125a-HG	5'-GGGCTCAGATTGCCAAGACA-3'	5'-GTTCTCCTCCACCCCATAGC-3'
mir-30c-HG	5'-AACCTGACAGGGAAAGCTCTG -3'	5'-CTCATCCCGAGTGACCTGTG-3'
mir-182-HG	5'-GGAAATGAGGTGGCCCTTGA-3'	5'-CCAGATGACCTCTGACCTGC-3'
mir-200c-HG	5'-AAGAAGGGGCTTCCAGGTTA-3'	5'-GGAAGTGTCCCAAATGACG-3'
let-7a-HG	5'-GAAATGTTTGTGTGTGGTAGTCAG-3'	5'-TCAGGGAATAAGGTTGCCTATCGC-3'
Mirc1	5'-TTGTTGCCCTCAGGACCTTG -3'	5'-CTTTCCGAGCCCCATTCCAA-3'
Mirc3	5'-GCCAGGTCAATAAGCCCAGT-3'	5'-GCCAGGTCAATAAGCCCAGT-3'
miR-106HG	5’-CTGAACGCCACCTTTACCCT-3’	5’-TCTTAGCTGTGGAAGGGGGA-3’
Mirc22	5’-CTCCATGTCTCCACAGTCGC-3’	5’-GTTCCTGCTGAACTGAGCCA-3’
miR-186HG	5’-GGGCCACTGAACCGTGAG-3’	5’-CCCTGACAAGAAGTGAGCGT-3’
miR-206HG	5’-AACAGCTGCCAGTGTCCATT-3’	5’-GCCCAGGCGCTATTGTACTT-3’
Aicda, Prdm1 and I_H ChIP		
Aicda promoter	5’-GGAGGCAGATGTTGGATACC-3’	5’-ATATCGGTCTCCAGCGTGAC-3’
Aicda region 4	5’-AAATCGGGGAATGCAGAAGT-3’	5’-TCCTCGGGTCACTATTTTTGG-3’
Prdm1 promoter	5’-ACTCCAGGACTACACAGCGA-3’	5’-GGATCGCTAGCTTCCTGTCG-3’
Iμ promoter	5’-TGAAACACTCTGTCCAGCCC-3’	5’-GAGGACCTCTCCAGTTTCGG-3’
Iγ1 promoter	5’-CCCCAGAATGAAGGGGAACC-3’	5’-CAGCCTTTGTCCCAGAGAGG-3’
Iγ3 promoter	5’-ATCCACGTGATGCAGCTTGA-3’	5’-AATGCTACCCCACACCCATC-3’
Iα promoter	5’-CGCATCTCTGTCTCAGGGTC-3’	5’-CTTGCCTCCACGTCATGTCT-3’
Somatic mutations		
$V_{186.2}DJ_H$-Cγ1	5’-CATGCTCTTCTTGGCAGCAACAGC-3’	5’-GTGCACACCGCTGGACAGGGATCC-3’
$V_{186.2}DJ_H$-Cγ2b	5’-CATGCTCTTCTTGGCAGCAACAGC-3’	5’-CGGAGGAACCAGTTGTATC-3’
$V_{186.2}DJ_H$-Cγ3	5’-CATGCTCTTCTTGGCAGCAACAGC-3’	5’-ACCAAGGGATAGACAGATGGGG-3’
